# Golgi organization is regulated by proteasomal degradation

**DOI:** 10.1038/s41467-019-14038-9

**Published:** 2020-01-21

**Authors:** Avital Eisenberg-Lerner, Ron Benyair, Noa Hizkiahou, Neta Nudel, Roey Maor, Matthias P. Kramer, Merav D. Shmueli, Inbal Zigdon, Marina Cherniavsky Lev, Adi Ulman, Jitka Yehudith Sagiv, Molly Dayan, Bareket Dassa, Mercedes Rosenwald, Idit Shachar, Jie Li, Yanzhuang Wang, Nili Dezorella, Suman Khan, Ziv Porat, Eyal Shimoni, Ori Avinoam, Yifat Merbl

**Affiliations:** 1 Department of Immunology, Weizmann Institute of Science, Rehovot, Israel; 2 Department of Molecular, Cellular and Developmental Biology, University of Michigan, Ann Arbor, MI USA; 3 Department of Chemical Research Support, Weizmann Institute of Science, Rehovot, Israel; 4 Department of Biomolecular Sciences, Weizmann Institute of Science, Rehovot, Israel; 5 Flow Cytometry Unit, Life Sciences Core Facilities, Weizmann Institute of Science, Rehovot, Israel

**Keywords:** Golgi, Proteolysis

## Abstract

The Golgi is a dynamic organelle whose correct assembly is crucial for cellular homeostasis. Perturbations in Golgi structure are associated with numerous disorders from neurodegeneration to cancer. However, whether and how dispersal of the Golgi apparatus is actively regulated under stress, and the consequences of Golgi dispersal, remain unknown. Here we demonstrate that 26S proteasomes are associated with the cytosolic surface of Golgi membranes to facilitate Golgi Apparatus-Related Degradation (GARD) and degradation of GM130 in response to Golgi stress. The degradation of GM130 is dependent on p97/VCP and 26S proteasomes, and required for Golgi dispersal. Finally, we show that perturbation of Golgi homeostasis induces cell death of multiple myeloma in vitro and in vivo, offering a therapeutic strategy for this malignancy. Taken together, this work reveals a mechanism of Golgi-localized proteasomal degradation, providing a functional link between proteostasis control and Golgi architecture, which may be critical in various secretion-related pathologies.

## Abstract (web summary)

Correct Golgi assembly is important to cellular homeostasis but regulation of its structure under stress remains unclear. Here, the authors identify stress-induced degradation of GM130 by Golgi-localized 26S proteasomes, leading to Golgi dispersal.

## Introduction

The Golgi apparatus is the central hub of intracellular protein sorting and trafficking. In vertebrates, the architecture of the Golgi apparatus is largely defined by tethered stacks of cisternae that are held together as a perinuclear Golgi ribbon^1^. The Golgi is a highly dynamic structure required to cope with membrane and protein influx and transport to support cellular trafficking. Such membrane dynamics allow adaptation to changing conditions of intracellular and extracellular environments such as cell division, increased load of secretory proteins, or wound healing^2,3^. It is thus not surprising that the structural organization of the Golgi ribbon is tightly regulated and is essential for the establishment of cell polarity, membrane traffic and accurate protein glycosylation of surface and secreted proteins^4–6^. Indeed, perturbations in Golgi architecture have been observed in various human diseases including Golgi congenital diseases, neurodegeneration and cancer^7–10^.

Key to maintaining Golgi organization are the group of Golgi tethering proteins that link the Golgi stacks to a ribbon^11–13^. One of the most studied tethering proteins is GM130, a component of the Golgi matrix that anchors adjacent stacks through interacting with GRASP65 at its C-terminus and p115 at its N-terminus^14,15^. Disrupting the GM130-p115 complex with competing antibodies or peptides, or through expression of GM130 mutants, inhibited Golgi assembly^14,16^. Likewise, experimental depletion of GM130 led to loss of the ribbon architecture into individual stacks^12,17,18^. Further, regulation of GM130 through phosphorylation was shown to facilitate Golgi disassembly during mitosis^14,19^. Interestingly, alterations in GM130 are reported in various pathologies. Brain-specific deletion of GM130 in mouse models caused Golgi fragmentation and cell death in Purkinje neurons, leading to neuronal loss and ataxia^18^. Dysregulation of GM130 was also shown to be involved in Golgi fragmentation in Alzheimer’s disease^20^. Finally, altered GM130 expression levels have been described in various cancers, in association with disruption of the Golgi structure and loss of cell polarity, leading to increased migration and invasion properties of the tumors. Interestingly, while these phenotypes were associated with loss of GM130 in breast and colorectal cancers^21,22^, increased invasion and poor prognosis are associated with high levels of GM130 in lung and gastric tumors^23,24^, suggesting that GM130 levels are tightly regulated and aberrations in its expression may have detrimental effects.

Recently, localized proteasomal degradation has emerged as an important cellular mechanism for organelle autoregulation and proteostasis control^25–29^. However, the potential involvement of the 26S proteasome in Golgi homeostasis and in response to Golgi stress was not characterized. We thus sought to examine a role for proteasomal degradation in Golgi autoregulation. We reveal that Golgi-specific stress provokes Golgi dispersal in a proteasome-dependent manner, via the degradation of GM130. Further, we show that the response to Golgi stress, which is independent of the canonical unfolded protein response (UPR), is mediated through PSMD6, a regulatory subunit of the proteasome together with p97/VCP. Importantly, we found that 26S proteasomes are localized to the cytosolic surface of the Golgi apparatus, enabling localized degradation and regulation of Golgi organization. Aberrations in Golgi-localized degradation disrupt the Golgi architecture and consequently lead to cell death in multiple myeloma cells, which are highly engaged in protein glycosylation and secretion. Lastly, our findings demonstrate that this pathway may be targeted to elicit detrimental Golgi stress and significant cytotoxicity of multiple myeloma cells in vitro and in vivo. Taken together, our findings suggest that Golgi-apparatus related degradation (GARD) may afford a novel paradigm by which the local concentration of Golgi tethering proteins is regulated by the ubiquitin-proteasome system to control Golgi homeostasis.

## Results

### Proteasomes regulate Golgi dispersal via GM130 degradation

**Fig. 1:**
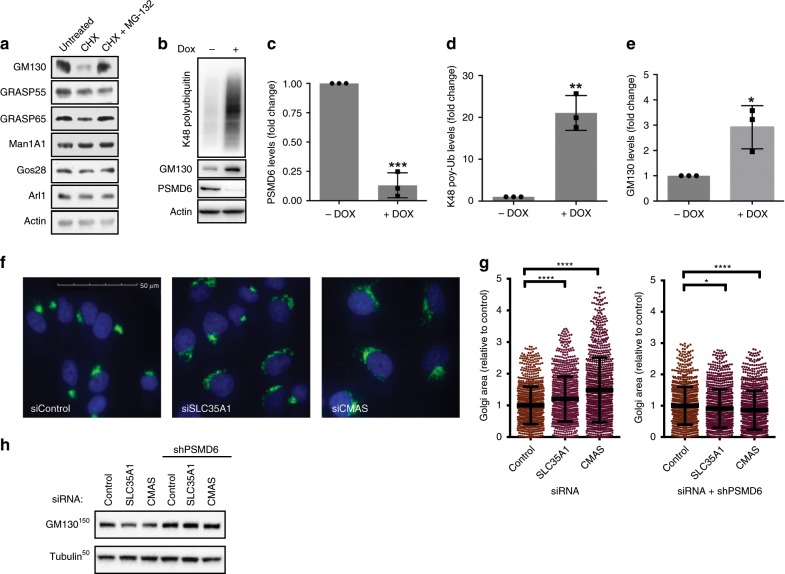
Golgi stress induces proteasomal degradation of GM130. **a** HeLa cells were treated with either cycloheximide (CHX 100 µg/ml, 8 h), CHX and MG-132 (20 µM, 8 h) or left untreated and analyzed by WB with the indicated antibodies. **b**–**e** HeLa cells expressing an inducible knockdown shRNA vector targeting PSMD6 were either treated with DOX to induce shRNA expression, or left untreated. **b** The expression levels of PSMD6, GM130, or K48-linked poly-ubiquitination were evaluated by WB analysis. Image is representative of three experiments. *p*(PSMD6) = 0.0001; *p*(K48) = 0.0011; *p*(GM130) = 0.0175. **c**–**e** Band intensities relative to actin were quantified using ImageLab. *n* = 3 independent experiments. Error bars = SD. *p*(PSMD6) = 0.0065, *p*(K48) < 0.0001,*p*(GM130) = 0.0039 (*t*-test, log relative intensity). **f**, **g** A549 cells stably expressing an inducible shRNA for PSMD6 were transfected with siRNA targeting wither SLC35A1, CMAS, or non-targeting control and incubated in the presence of either DOX (to induce PSMD6 shRNA) or DMSO as control for 72 h. **f** Representative images of Giantin immunostaining in the different cells. Scale bar = 50 μM. **g** The relative Golgi size was determined based on Giantin staining. Each dot represents a single cell. Error bars = SD. *n* = 1000 cells; *p = 0.0123; ****p < 0.0001 (one way ANOVA with Sidak’s multiple comparisons test). **h** Cells treated as in F were analyzed by WB for GM130 expression. Source data are provided as a Source Data file.

To examine the potential involvement of proteasomal degradation in regulating the Golgi organization, we first examined the half-life of various Golgi-localized proteins in response to proteasome inhibition. We found that the levels of the Golgi tethering factors GM130 and GRASP65 were reduced following 8 h of translation inhibition, but were stabilized in the presence of a proteasome inhibitor (Fig. 1a). In contrast, the levels of non-structural transmembrane Golgi proteins such as Man1A1 and Gos28 did not change during this time (Fig. 1a). To further examine a role for the 26S proteasome in degradation of tethering proteins, we focused on GM130 and monitored its levels in A549 isogenic cells that express an inducible shRNA of PSMD6, a 19S regulatory particle subunit, leading to its controlled knockdown (Fig. 1b, c). Silencing of PSMD6 expression led to a significant increase in the protein levels of GM130 that was accompanied by a general increase in general K48-specific poly-ubiquitination (Fig. 1b–e).

Based on these findings, we hypothesized that proteasomal degradation of GM130 might offer a mechanism to control Golgi morphology in response to changes in cellular conditions. It was previously shown that inhibition of terminal Golgi glycosylation (i.e., fucosylation and sialylation) causes the accumulation of glycoconjugated-proteins at the Golgi, that in turn lead to changes in the Golgi structure^30^. We therefore asked whether Golgi organization may be directly regulated under stress conditions, such as impairment of terminal glycosylation, by GM130 degradation. To impair terminal glycosylation, we knocked-down the expression of either SLC35A1, a Golgi sialyltransferase, or CMP-Neu5Ac Synthetase (CMAS), which catalyzes the synthesis of CMP-sialic acid, in the background of the inducible PSMD6 knockdown. Silencing of either SLC35A1 or CMAS (Supplementary Fig. 1A, B) disrupted Golgi morphology and led to an increase in Golgi size (Fig. 1f, g, left). Importantly, knockdown of either SLC35A1 or CMAS led to the downregulation of GM130 (Fig. 1h). However, inducing the knockdown of PSMD6, which prevented the proteasomal degradation of GM130 (Fig. 1h), successfully rescued the Golgi dispersal otherwise induced by the depletion of SLC35A1 or CMAS (Fig. 1g, right). These findings suggest that impaired terminal glycosylation at the Golgi induces proteasome-dependent degradation of GM130 and disruption of Golgi organization.

**Fig. 2:**
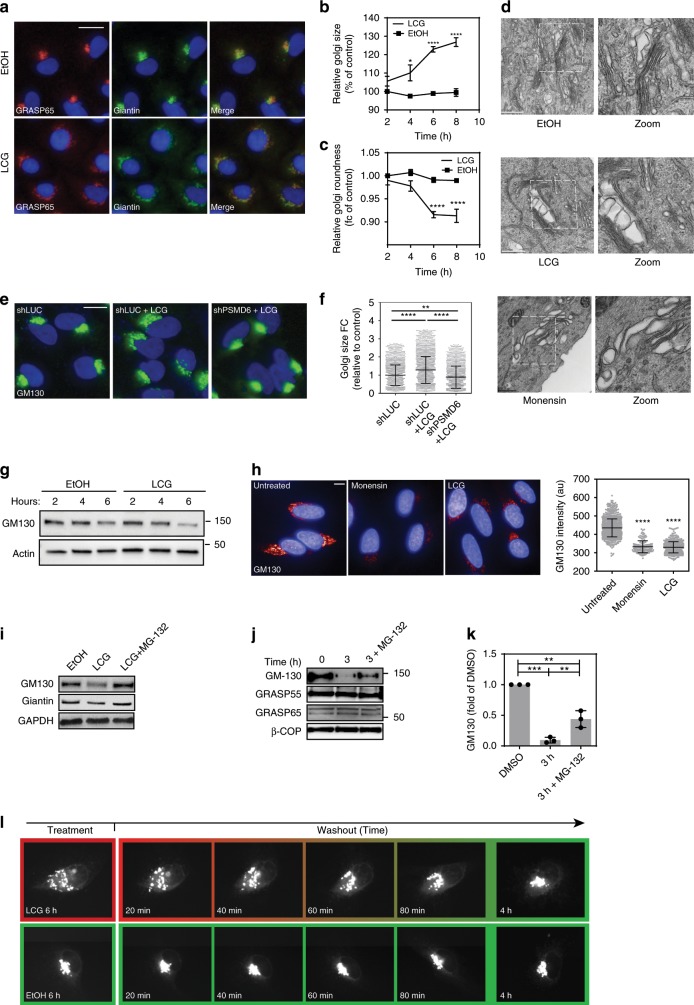
The 26S proteasome is required for dispersal of the Golgi apparatus. **a** A549 cells treated with LCG or EtOH for 8 h. *n* = 5900 cells in three independent experiments. GRASP65 (green); Giantin (red); Nuclei (blue). Scale bar = 20 μM (**b, c**) Golgi size (**c**) and roundness (**d**) of cells treated as in **a** were measured based on GRASP65 staining relative to EtOH, 2 h. *n* = 3 independent experiments, 6790 cells on average/condition. *p* = 0.0001 (Dunnet multiple comparisons test). Error bars = SEM. **d** A549 cells were treated with LCG (200 μM), monensin (2 μM), or control for 6 h and imaged by transmission electron microscopy. **e**, **f** HeLa cells expressing shRNA targeting either PSMD6 or luciferase (shLuc) were treated with LCG or EtOH. **e** Representative images. GM130 (green); Nuclei (blue). Scale bar = 20 μM. **f** The relative Golgi size compared to control was determined based on GM130 staining. FC: fold change. *n* = 1687 cells on average/condition. Error bars = SD. **p = 0.0082; ****p < 0.0001 (one way ANOVA with Sidak’s multiple comparison test). **g** RPMI-8226 cells were treated with cycloheximide (CHX 100 µg/ml) and either LCG (200 µM) or EtOH for the indicated times, and analyzed by WB with the indicated antibodies. Image is representative of three experiments. Provided also as a Source Data file. **h** HeLa cells were treated with either Monensin (2 µM) or LCG (200 µM) and stained for GM130. Left: representative cell images. The intensity of GM130 per cell was analyzed using Harmony software. *n* = 500 cells per condition. *p* < 0.0001 (one way ANOVA). **i** RPMI-8226 cells were treated with LCG (200 µM), LCG (200 µM) + MG132 (20 µM) or EtOH as control for 4 h, and analyzed by WB with the indicated antibodies. *n* = 3 experiments. **j** HeLa cells were fractionated on sucrose cushions and Golgi-enriched fractions were incubated for either 0 or 3 h at 37 °C and supplemented with MG-132 (20 µM) where indicated. Image is representative of three experiments. **k** Quantification of H. *n* = 3 independent experiments; Error bars = SD; ***p < 0.001, ****p < 0.0001 (one-way ANOVA with Holm-Sidak’s multiple comparison test). **l** HeLa cells expressing GalT-YFP were treated with LCG (200 µM) or EtOH for six hours, then washed three times with medium and imaged in 20 min intervals for 4 h.

The above findings prompted us to more broadly investigate the involvement of proteasomes in regulating Golgi organization in response to various Golgi-stress conditions^9^. We sought to exploit pharmacological perturbations that were previously described to induce Golgi stress, which in contrast to constitutive genetic manipulations, allow evaluating the early and direct effects of the Golgi stress response. Specifically, we analyzed the Golgi morphology in the inducible PSMD6 knockdown cells following their treatment with either CMP-3F-NeuAc, an inhibitor of sialylation^31^, or with nigericin, an antiporter of protons and potassium ions that causes Golgi stress and fragmentation^32^, in comparison to Brefeldin A (BFA) that causes the redistribution of the Golgi into the ER via inhibition of ARF1^33^. Indeed, we found that both CMP-3F-NeuAc and nigericin disrupt Golgi organization as evident by the increased Golgi size following treatment (Supplementary Fig. 1C, D). This change in Golgi morphology occurred in a proteasome-dependent manner as reflected in the ability of PSMD6 knockdown to rescue this effect (Supplementary Fig. 1E, F). In contrast, redistribution of the Golgi into the ER by BFA was independent of proteasome activity (Supplementary Fig. 1G, H). To corroborate these findings further, we also examined whether proteasome activity regulates the response to two additional known stressors, namely, monensin, an ionophore that neutralizes luminal pH and blocks intra-Golgi trafficking^34–39^, and lithocholylglycine (LCG/Lith-Gly), which belongs to the family of lithocholic acid analogs commonly used to inhibit 2–3 linkage sialyltransferase activity^40–43^. We confirmed that LCG altered protein sialylation as evident by the reduced secretion of sialylated proteins, using secretome protein enrichment with click sugars (SPECS)^44^ (Supplementary Fig. 2A). Next, we analyzed the Golgi size and found that treatment of cells with either monensin or LCG led to a significant increase in the average Golgi size, as early as 4 h after treatment (Fig. 2a–c and Supplementary Fig. 2B). We further demonstrated the disruption of the Golgi apparatus in response to LCG or monensin treatments using transmission electron microscopy (Fig. 2d). Importantly PSMD6 deficiency blocked Golgi dispersal in response to stress, significantly reducing Golgi size (Fig. 2e, f).

Consistent with the findings that GM130 is destabilized in SLC35A1 and CMAS deficient cells, we found that the half-life of GM130 was reduced by monensin and even more so by direct inhibition of sialylation with LCG (Fig. 2g, h and Supplementary Fig. 2C). Inhibition of the proteasome abrogated the stress-induced degradation of GM130 under Golgi stress, both in intact cells (Fig. 2i), and in vitro in Golgi-enriched fractions separated on sucrose gradients (Fig. 2j, k and S2D, E). We further showed that ectopic expression of GM130, which mimics the impact of proteasome inhibition, attenuates Golgi dispersal under stress (Supplementary Fig. 2F, G). Finally, by examining Golgi organization in time-lapse movies in response to stress, we demonstrate that washing out of LCG results in re-assembly of the Golgi within 4 h of drug removal (Fig. 2l and Supplementary Fig. 2H), suggesting that restoring Golgi morphology may be advantageous for the cell in the absence of an insult. Taken together, these findings indicate that changes in Golgi integrity imposed by Golgi stress are dynamic and reversible, and are actively regulated by cellular proteasomes.

### 26S proteasomes localize to the cytosolic surface of the Golgi

**Fig. 3:**
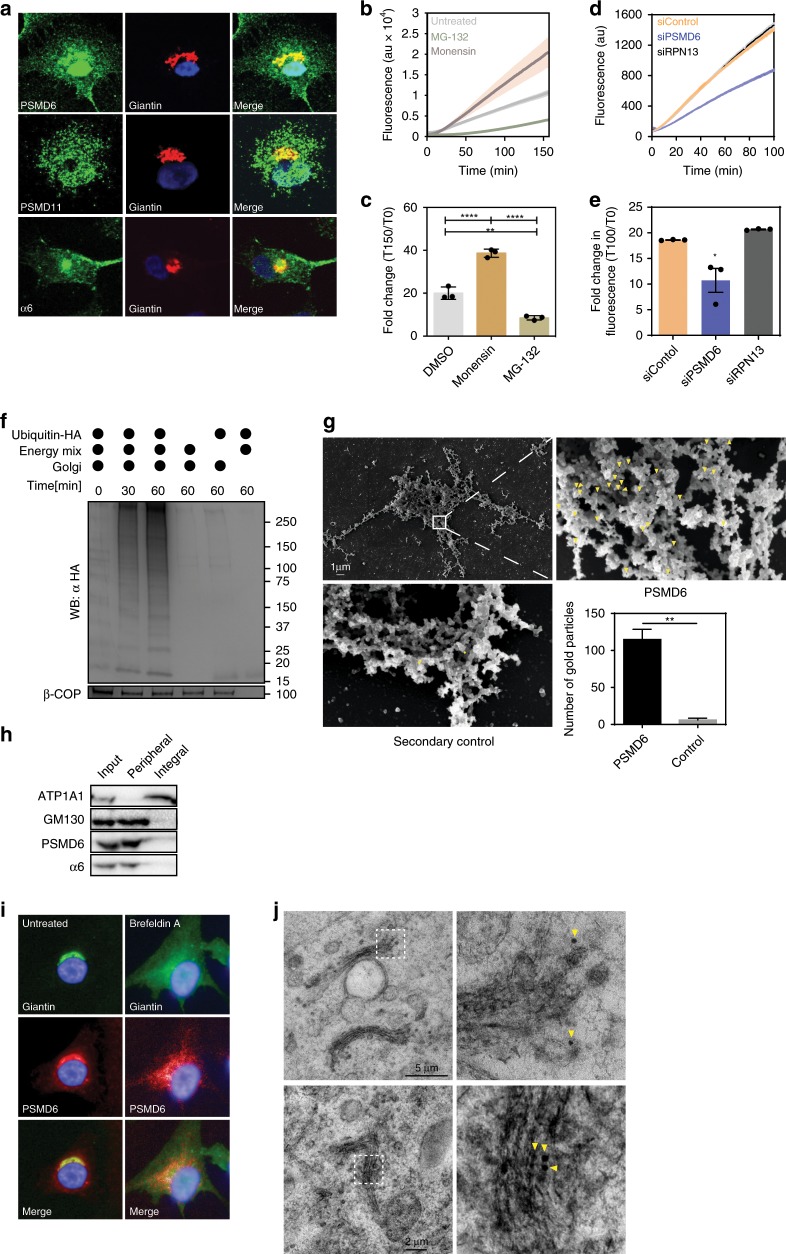
26S proteasomes are localized to the cytosolic surface of the Golgi apparatus. **a** Confocal immunofluorescence images of the proteasome subunits PSMD6, PSMD11, and Alpha 6 (Green), the Golgi marker Giantin (red), and nuclei (blue) in A549 cells. Cells were permeabilized by digitonin prior to fixation. Images are representative of >30 cells. **b**, **c** Suc-LLVY-AMC proteasomal degradation assay of Golgi fractions from cells treated with monensin or MG-132 or untreated. Colored backgrounds represent SEM. **c** Quantification of the fold increase in AMC fluorescence at the final measurement (*t* = 150) from the initial time-point (*t* = 0) ± SEM. *n* = 3 independent experiments; *p*(untreated) = 0.002; *p*(monensin) = 0.01; *p*(MG-132) = 0.002 (one way ANOVA with Tukey’s multiple comparison test). **d**, **e** Suc-LLVY-AMC proteasomal degradation assay of Golgi-enriched fractions from cells in which the expression of either PSMD6, RPN13 or control were knocked down by specific siRNAs. Standard error shown as faded area around primary line. *n* = 3 biologically independent repeats. **e** Quantification of the fold increase in AMC fluorescence at the final measurement (*t* = 100) from the initial time-point (*t* = 0). *n* = 3 independent experiments. Error bars = SD. *p*(siControl) = 0.0004; *p*(siPSMD6) = 0.0001; *p*(siRPN13) = 4.13 × 10^−5^ (one way ANOVA with Tukey’s multiple comparisons test). **f** Purified Golgi fractions were supplemented with ATP and HA-ubiquitin (HA-ub) and incubated for 0, 30, and 60 min at 37 degrees. β-COP was used for normalization. Image is representative of three experiments. Source data are provided as a Source Data file. **g** Scanning electron microscopy images of Golgi-enriched fractions that were immuno-gold labeled with antibodies against either PSMD6 (inset) or primary antibody control (bottom). Arrowheads mark labeled spots. Images are representative of two independent experiments. Graph shows the quantification of gold particles in samples labeled with antibodies against either PSMD6 or control. *n* = 3 measurements from two independent experiments. Error bars = SD. *p* = 0.0015 (unpaired two-tailed *t*-test). **h** Golgi-enriched fractions were washed in alkali buffer to dissociate peripheral proteins. Images are representative of two experiments. **i** Immunofluorescence images of HeLa cells treated with Brefeldin A (where indicated). Giantin (Green); PSMD6 (red). Images are representative of >30 cells. **j** Immuno-gold labeling of PSMD6 after high pressure freezing. Insets = zoom in on gold particle labeling at the Golgi, marked by yellow arrowheads. Images are representative of two experiments.

The degradation of Golgi tethering factors that are tightly bound to the Golgi membrane suggested that there might be a requirement for proteasomes that are localized within the close vicinity, or associated with, the cytosolic surface of the Golgi membrane. Previous studies have reported that proteasomes associate with membranes of the endoplasmic reticulum via the 19S particle of the proteasome^27,45^. We therefore hypothesized that proteasomes might associate with Golgi membranes in a similar manner. Interestingly, we found subunits of both the 19S regulatory particle and the 20S core particle to localize to the Golgi apparatus using both immunofluorescence and cellular fractionation by sucrose gradients (Fig. 3a and Supplementary Fig. 3A). Affinity purification of PSMD6 or pull down using Ubiquitin Interacting Motif (UIM)-GST from Golgi-enriched fractions, coupled to mass-spectrometry, identified both the 19S and 20S particles as well as numerous components of the ubiquitin proteasome system (Supplementary Fig. 3B–D, Supplementary Data 1).

Prominently, we demonstrated using proteasome activity assays^46^ that subcellular Golgi-enriched fractions contained active proteasomes and that proteasome activity was increased upon Golgi-stress (Fig. 3b, c). In agreement with the requirement of the 19S particle for Golgi dispersal upon stress, we observed reduced proteasomal activity in Golgi-enriched fractions that were produced from cells knocked-down in PSMD6, a stoichiometric 19S subunit. In contrast, knockdown of a non-stoichiometric regulatory subunit, RPN13, did not affect the proteolytic activity in these samples (Fig. 3d–e). Based on this, we asked whether the Golgi compartment may autonomously facilitate not only proteasomal degradation, but also the canonical upstream event of poly-ubiquitination. Using in vitro ubiquitination assays, we found that de novo poly-ubiquitination may be executed autonomously in Golgi-enriched subcellular fractions (Fig. 3f).

To assess the physical association of proteasomes to Golgi membranes, we examined PSMD6 by immuno-gold staining of isolated Golgi membranes, using scanning electron microscopy (Fig. 3g; yellow arrowheads). As these fractions were purified without the use of membrane-permeabilizing detergents, the immuno-gold labeling of PSMD6 suggests that PSMD6 is bound to the cytosolic surface of the Golgi membrane. We further confirmed this finding using an alkali wash assay that removes non-integral proteins from the membrane, demonstrating that proteasomes were associated with Golgi membranes, rather than integrated into them (Fig. 3h). In addition, BFA treatment led to dispersal of perinuclear PSMD6, supporting its localization at the Golgi (Fig. 3i and Supplementary Fig. 3E). Finally, to corroborate these results in intact cells, we utilized high pressure freezing and immuno-gold labeling of PSMD6 in transmission electron microscopy, and demonstrated the precise localization of proteasomes to Golgi stacks (Fig. 3j).

### Proteasomal degradation of GM130 is mediated by p97/VCP

**Fig. 4:**
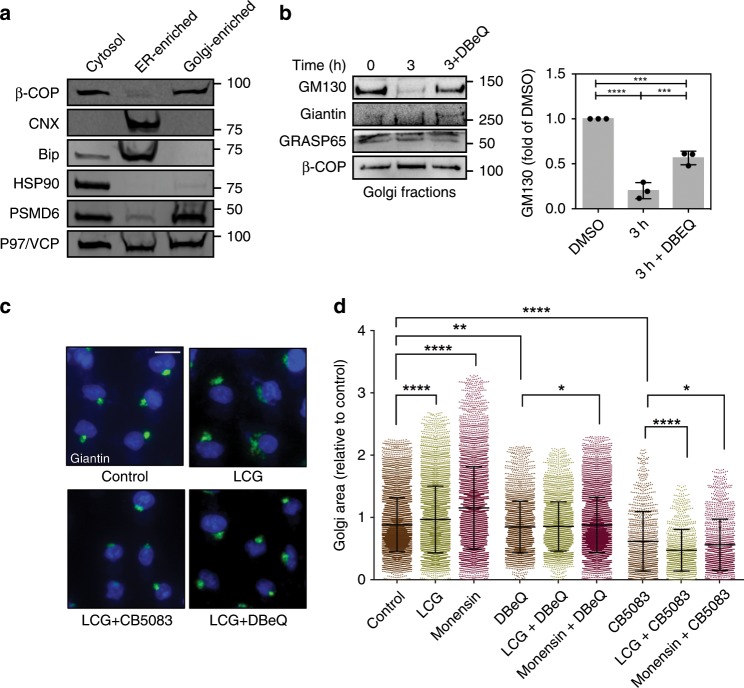
Localized degradation of GM130 is mediated by p97/VCP and 26S proteasomes. **a** Western blot analysis of various subcellular organelle markers following sucrose cushion fractionation (see Methods for details). Image is representative of >3 experiments. **b** Golgi-enriched fractions were supplemented with DBeQ (10 µM) where indicated and incubated for three hours at 37 °C. Top: Representative WB. Bottom: Quantification of three experiments. Source data are provided as a Source Data file. **c**, **d** A549 cells were treated with either LCG (200 μM), monensin (2 μM), or control, in the presence of either DBeQ (1 μM), CB-5083 (0.5 μM), or DMSO as control, for 6 hours. Cells were then fixed and stained with anti Giantin antibodies. **c** Representative cell images. Scale bar = 20 μM. **d** Golgi area was measured and calculated relative to control cells. *n* > 1000 cells. *p(DBeQ vs. DBeQ + mon) = 0.0121; *p(CB5083 vs. CB5083 + mon) = 0.0261; **p (DBeQ vs. control) = 0.0086; ****p < 0.0001 (one-way ANOVA with Sidak multiple comparisons test).

In accordance with the role of p97/VCP in targeting substrates to proteasomal degradation in other cellular organelles such as the ER and mitochondria^47–49^, we hypothesized that p97 might be required for mediating the degradation of tethering factors that are tightly bound to the Golgi membrane. Consistent with previous reports^50,51^, we found abundant p97/VCP protein expression in Golgi-enriched fractions (Fig. 4a). We further showed that specific inhibition of p97/VCP with DBeQ blocked GM130 degradation in Golgi-enriched fractions (Fig. 4b). In addition, both DBeQ and CB5083 blocked Golgi dispersal under LCG and monensin treatments (Fig. 4c, d). Collectively, these results underscore the roles of p97/VCP and the 26S proteasome in targeting GM130 to Golgi apparatus-related degradation (GARD).

### Golgi stress induces cell death in multiple myeloma cells

**Fig. 5:**
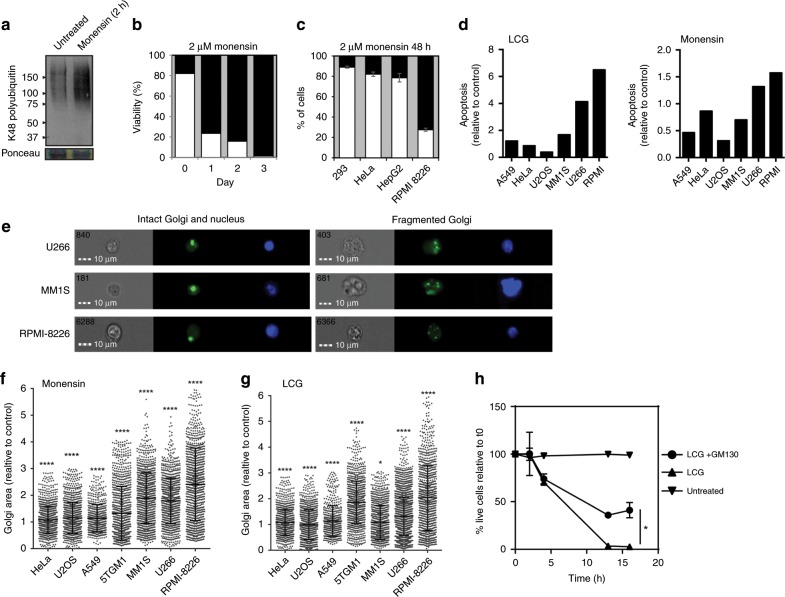
Golgi stress-induced death of Multiple myeloma cells is GM130-dependent. **a** Western blot analysis of K48-linked polyubiquitin chains in Golgi-enriched fractions collected from RPMI-8226 cells either untreated or treated with monensin for 2 h. Images are representative of three experiments. **b** Live/dead cell count of the indicated cell lines following 48 h of monensin treatment (2 µM). *n* = 2 independent experiments. **c** Live/dead cell count of RPMI-8226 cells over three days of treatment with monensin (2 µM). *n* = 2 independent experiments. **d** The indicated cell lines were treated with either LCG (200 μM, left) or monensin (2 μM, right), for 6 h and stained for nuclei by Hoechst. Apoptotic cells were determined based on nuclear morphology and DNA condensation in Imaging Flow Cytometer ImageStreamX mark II (mm cell lines). *n* > 25,000 cells. **e**–**g** The indicated cell lines were treated with either LCG (200 μM), monensin (2 μM) or control for 6 h and imaged by Imaging Flow Cytometer ImageStreamX mark II (Multiple myeloma cell lines) or fluorescent microscopy (adherent cell lines) for Golgi by Giantin staining and nuclei by Hoechst. **e** Shown are representative images of cells with intact or fragmented nuclei. **f**, **g** Golgi area of cells treated with monensin (**f**) or LCG (**g**) was measured and calculated relative to control cells. Error bars = SD. *n*(LCG) = 9668 cells. *n*(monensin) = 7183 cells. *p = 0.019; ****p < 0.0001 (Bonferroni multiple comparisons one way ANOVA test). **i** RPMI-8226 cells were either untreated or treated with LCG (200 µM) either with or without transfection to ectopically express GM130. Live cells were counted based on trypan blue exclusion in the indicated times after treatment. Error bars = SD. *n* = 2 independent experiments. *p* = 0.0223 (unpaired two tailed *t*-test, 16 h).

Next, we sought to examine the functional consequences of inducing Golgi stress in highly secretory cells, which are heavily reliant on proper Golgi function. To that end, we chose to focus on multiple myeloma (mm), as this is a plasma cell malignancy characterized by a heavy load of glycoprotein production and secretion (e.g., antibodies). Within two hours of monensin treatment to mm (RPMI-8226) cells, we could see activation of the ubiquitin-proteasome system as evident by the high increase of K48-linked polyubiquitination that was detected (Fig. 5a). We found that prolonged treatment with monensin was toxic to mm cells, leading to cell death within three days of treatment in culture (Fig. 5b). Interestingly, we found that Golgi stress-induced toxicity was substantially more prominent in mm cells (Fig. 5c, d, Supplementary Fig. 4A) and in accordance provokes a significantly larger increase in Golgi size, compared to other cancer cell lines (Fig. 5e–g and Supplementary Fig. 4B). Importantly, ectopic expression of GM130 increased the viability of mm cells treated with LCG (Fig. 5h and Supplementary Fig. 5A), suggesting GM130 plays a protective role in the sensitivity to Golgi stress and apoptosis. The CCAAT-enhancer-binding protein homologous protein (CHOP) has been shown to promote cell death upon activation in numerous cellular stress conditions^52,53^. Indeed, we confirmed that Golgi stress led to upregulation of CHOP (Supplementary Fig. 5B), suggesting its potential involvement in Golgi stress-induced cell death. In agreement with previous reports^35,37,38^, CHOP activation under Golgi stress was independent from the canonical unfolded protein response as evident by the upregulation of Bip and ATF4, the splicing of XBP1, and the cleavage of activating transcription factor 6 (ATF6)^54,55^, only in response to treatment with tunicamycin, but not monensin or LCG (Supplementary Fig. 5C–E).

### A physiological role for Golgi stress in malignancy

**Fig. 6:**
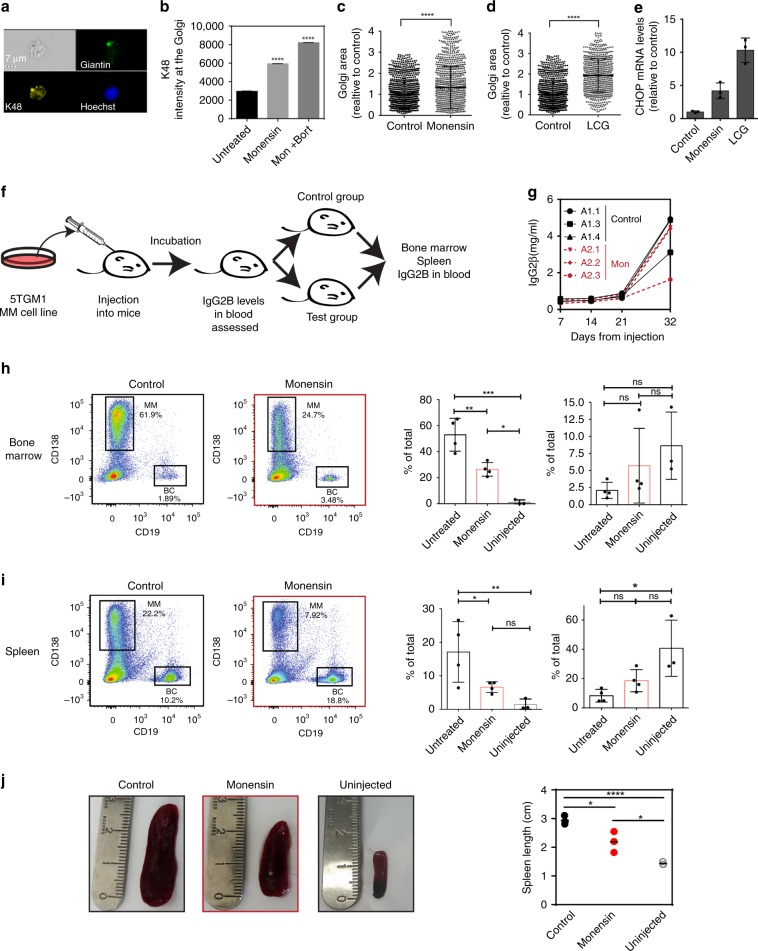
Golgi stress is toxic to Multiple myeloma cells in vivo. **a**, **b** 5TGM1 cells were treated with monensin (2 µM, 6 h) and where indicated Bortezomib (40 nM) was added for the last 2 h of incubation. Cells were imaged by the Imaging Flow Cytometer ImageStreamX mark II using anti-GRASP65 antibodies, anti K48-TUBE and Hoechst. K48 intensity was calculated per the Golgi area based on GRASP65 localization. A representative image is shown in **b**. *n* = 11,872 cells. Error bars = SD. *p* < 0.0001 (one-way ANOVA with Tukey’s multiple comparisons test). **c**, **d** 5TGM1 cells were treated with either LCG (200 μM), monensin (2 μM), or control for 6 h and imaged by Imaging Flow Cytometer ImageStreamX mark II for Golgi using GRASP65 antibodies and nuclei by Hoechst. Golgi area was measured in cells treated with either monensin (**c**) or LCG (**d**) and calculated relative to control cells. Error bars = SD. n = 3076 cells. *p* < 0.0001(Bonferroni multiple comparisons one way ANOVA test). **e** 5TGM1 cells were treated with either LCG (200 μM), monensin (2 μM), or control for 6 h and CHOP mRNA levels were determined by qPCR. *n* = 3 independent biological repeats. Error bars = SD. *p*(monensin) = 0.0098; p(LCG) = 0.0009 (two tailed *t*-test). **f** Schematic outline of the 5TGM1 mm mouse model experiment. **g** Blood IgG2β levels were measured by ELISA from mice injected with mm5TGM1 cells over a period of 32 days. Test mice were split into two groups, A1 (control) and A2 (monensin treated) and labeled individually as A1.1–4 and A2.1–4. Individual mice A1.2 and A2.4 died before treatment could begin. **h** FACS analysis and quantification of mm (CD138+) and normal B cells (CD19+) in bone marrow of control vs. monensin-treated mice. *n* = 12 mice. Error bars = SEM. *p*(mm untreated vs. monensin) = 0.008. (One-way ANOVA with Tukey’s multiple comparison test) **i** FACS analysis and quantification of mm cells and normal B cells (BC) in spleens of control vs. monensin-treated mice. *n* = 12 mice. *p*(mm untreated vs. monensin) = 0.001 (one way ANOVA with Tukey’s multiple comparison test)**. j** Spleen images and quantification of sizes. *n* = 12 mice. Error bars = SEM. ns: non-significant. *p < 0.05; ****p < 0.0001 (One-way ANOVA with Tukey’s multiple comparison test).

Lastly, we wished to examine whether inducing Golgi stress may be toxic to mm cells in vivo. The 5TGM1 transplanted C57BL6/KaLwRij mouse model is widely used to study murine mm as it recapitulates many features of the human disease, such as monoclonal paraprotein as well as bone lesions^56^. We first characterized the response of murine 5TGM1 cells to Golgi stress ex-vivo. As observed for human RPMI-8226 cells above, 5TGM1 cells exhibited a significant increase in K48-polyubiqutin chains, in response to two hours of monensin treatment (Fig. 6a, b). Treatment of mice 5TGM1 cells with monensin or LCG induced Golgi dispersal and CHOP induction (Fig. 6c–e). Accordingly, CHOP expression was up-regulated upon monensin or LCG treatment in 5TGM1 cells (Fig. 6e).

To investigate the physiological response of mm cells to Golgi stress, we injected mice with 5TGM1 cells and monitored blood levels of IgG2B and the development of bone lesions to ascertain mm disease progression^56^ (Fig. 6f, g and Supplementary Fig. 6A). Since the half-life of LCG is limited to a few minutes in the blood^57^, we focused primarily on monensin treatment for in vivo characterization. Mice that developed mm, based on the criteria described above, were administered with monensin in the drinking water for 5 days. Remarkably, treatment of mm mice with monensin led to a significant decline in the levels of mm CD138+ cells in the bone marrow (62–25%; Fig. 6h, Supplementary Fig. 6B), and this phenomenon was likewise evident in the spleen (22–8%; Fig. 6i). In contrast, monensin did not affect the levels of B cells or T cells, supporting the premise that the highly secretory mm cells are more sensitive to monensin-induced toxicity (Supplementary Fig. 6C). In addition, prolonged administration of monensin in the drinking water did not show adverse effects in the phenotypes or weight of healthy mice (Supplementary Fig. 6D). Notably, the splenomegaly exhibited by untreated control mice was reduced by ~30% in mice that received monensin (Fig. 6j), providing evidence of a beneficial outcome of monensin treatment for the splenomegaly, which is often associated with mm.

## Discussion

The Golgi apparatus has been suggested as a sensor of stress and regulator of cell death processes^9^. A Golgi-stress response has been described in several systems depicting transcriptional changes in expression of the chaperone HSP47, transcription factors such as TFE3 and ETS whose target genes are related to spliceosome function and cell death induction, as well as Golgi structural genes such as GCP60^34,36,37,58–61^. Yet, the underlying mechanisms responsible for controlling Golgi morphology in response to stress remain poorly understood. Here, we report a role for the ubiquitin-proteasome system in Golgi organelle autoregulation and show that localized proteasomal degradation is required for dispersal of the Golgi apparatus under stress. While additional mechanisms have been suggested to regulate the levels of Golgi tethering factors and the Golgi morphology^62–65^, our observations support a role for the ubiquitin-proteasome system in this process. We find that even partial degradation of GM130 is sufficient to compromise Golgi organization as in all of the conditions tested we did not observe complete loss of GM130 in the cell. This is not surprising in light of the clear significance of GM130 in maintaining the Golgi ribbon.

The discovery of Golgi membrane-associated proteasomes provides an elegant mechanism by which localized degradation of tethering proteins may be tightly regulated to rapidly control Golgi structure and function. Notwithstanding the importance of the localized degradation of GM130, additional tethering factors may be subjected to proteasomal degradation under different signals to provide fine-tuned regulation of Golgi organization. Further, our findings merit further investigation of the full range of cellular substrates, as well as the enzymatic machinery, that are involved in the regulation of this pathway under various physiological conditions.

Our observation that p97/VCP facilitates the degradation of GM130 suggests its potential involvement in extraction of peripheral or integral membrane proteins at the Golgi. Therefore, p97/VCP and Golgi-localized proteasomes may potentially be involved in quality control processes, as was previously reported in ER or mitochondria-associated degradation mechanisms^47,66–68^. Whether and how transmembrane or luminal proteins may also serve as targets for cytosolic proteasomes, and if so how they may translocate across the Golgi membrane, is intriguing and remains to be determined in future studies. Nevertheless, the discovery of GARD suggests that proteasomes may be involved in post-ER quality control mechanisms, in mammalian cells.

Until recently, Golgi quality control was described to involve two potential arms, retrieval back to the ER, or targeting to vacuolar/lysosomal degradation (reviewed in the ref. ^69^). A Golgi quality control mechanism has been described as a distal checkpoint to sort aberrant proteins following exit from the ER^70–74^. A recent study by Crews and colleagues directly identified a protein misfolding response at the Golgi^58^. In yeast, misfolded or damaged proteins at the Golgi were shown to undergo ubiquitination by the DSC/Tul1 E3 ligase complex, which targets them to vacuolar degradation^70,75,76^. Interestingly, while this manuscript was under review, a role for the DSC complex in targeting membranal Golgi and endosome proteins to degradation by cytosolic proteasomes was described in S. Cerevisiea^77^. Collectively, these recent findings, together with our data, define a proteasome-dependent post-ER checkpoint in the secretory pathway. While the full range of functions of Golgi-localized proteasomes is yet to be determined, it is expected that multicellular organisms which maintain high order of Golgi organization, will have additional regulatory mechanisms that may be QC independent, at the Golgi. We envisage that GARD may also impact Golgi structure and function, in post-ER QC processes, but these remain to be investigated in future studies.

Golgi fragmentation has been described under different stress conditions and during apoptosis^62–65^ and is correlated with pathology^9,10^. Indeed, our observations indicate that perturbing Golgi homeostasis is toxic to mm cells in vitro and in vivo, highlighting monensin as a potential treatment for this malignancy that is orthogonal to current therapeutic approaches. We hypothesize that Golgi-stress induced cytotoxicity will depend on the glycoprotein production load of different cancer types. Intriguingly, perturbations of proteostasis in mm cells is already common practice in clinical settings as treatment with proteasome inhibitors (e.g., Bortezomib) is among the main therapeutic modalities offered in this disease^78^. Bortezomib toxicity is by large attributed to the inhibition of ERAD, required to relief the burden of massive Ig production^79^. Our results suggest that in addition to the ER, Golgi-localized proteasome activity may provide potential explanation for the clinical efficacy of proteasome inhibitors in mm treatment. Further, reduced levels of 19S subunits of the proteasome have been recently associated with resistance of mm cells to proteasome inhibitors^80–82^. Our finding, of PSMD6 as a key regulator of GARD, supports this notion and suggests that different regulatory subunits of the proteasome may have specialized physiological roles.

**Fig. 7:**
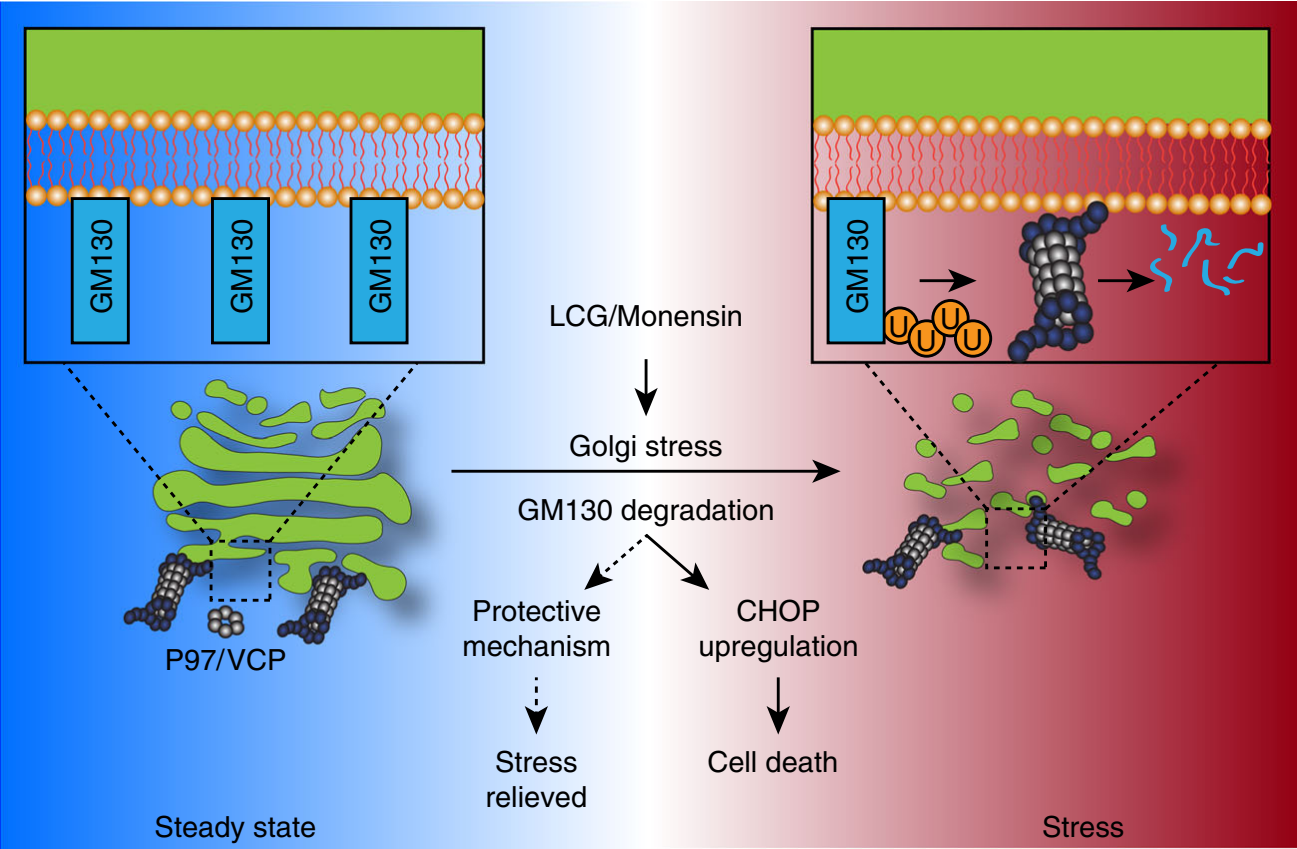
Golgi organization is regulated by proteasomal degradation. Proteasomes are constitutively associated with the Golgi membranes and mediate the degradation of the Golgi tethering protein GM130. Golgi-apparatus related degradation (GARD) is activated in response to Golgi-stress, such as block of sialylation by LCG, and induces enhanced degradation of GM130, leading to dispersal of the Golgi apparatus, upregulation of CHOP, and cell death. Accurate morphology of the Golgi may be retained upon relief of the stress (dotted line), suggesting that proteasome-dependent control of Golgi organization might serve as a cell-fate dictating mechanism during the stress-response.

It is likely that the consequences of aberrant protein load or misglycosylation at the Golgi will be time- and cargo-specific, and the full extent of the ubiquitin-proteasome system in regulation of Golgi homeostasis is yet to be determined. Here, we show that proteasome-mediated dispersal of the Golgi is actively regulated and may be reversed, and therefore could provide a temporal window to assess the cellular response to stress, and if required, signal to initiate apoptosis via CHOP activation. Indeed, the observed reorganization of the Golgi upon removal of the stress signal suggests that maintaining high-order organization of the Golgi is preferred (Fig. 7). Furthermore, although prolonged Golgi stress may induce ER stress, as was demonstrated in the case of constitutive inhibition of terminal glycosylation^30^, our findings suggest that an early, Golgi-specific, response to stress is sufficient to initiate cell death processes. It is plausible that cells have evolved surveillance mechanisms to respond to perturbations in Golgi homeostasis before they become toxic. Thus, GARD may serve as a ‘guardian’ of the Golgi apparatus under conditions in which the load imposed on this organelle overwhelms its capability to handle sorting, trafficking, or the fidelity of its structure.

Taken together, our findings elucidate a role for the ubiquitin-proteasome system in maintaining Golgi homeostasis and afford a novel paradigm by which GARD may have a role in regulating the cellular response in various physiological conditions associated with aberrant glycoprotein production, high load of secretion or protein aggregation^83–86^ and may bear clinical importance in diseases such as autoimmunity, inflammation, neurodegeneration and cancer.

## Methods

### Antibodies and plasmids

Mouse anti β-COP G6160 1:1000, Rabbit anti PSMD6 HPA036922 1:1000, Mouse anti HA H3663 1:1000 (Sigma), Rabbit anti Giantin, Rabbit anti TGN46, Mouse anti Giantin ab37266 1:1000, WB 1:100 IF, Giantin ab80864 1:200 IF, Rabbit anti GM130 ab52649 1:5000 WB, 1:200 IF, Rabbit anti PSMD6 ab140461 1:200, IF 1:4000 WB, Rabbit anti PSMD11 ab99414 1:5000, Mouse anti P97/VCP ab11433 |1:2000, Mouse anti 58K-Golgi protein ab27043 1:1000, Mouse anti Grp78 (Bip) ab181499 1:1000, Mouse anti GAPDH AB-AM4300 1:50,000, Rabbit anti Hsp90 ab13495 1:1000, Rabbit anti K48 linked polyubiquitin ab140601-100 1:5000, Rabbit anti Calnexin ab22595 1:1000, Rabbit anti PSMD11 (Cell signaling). Mouse anti alpha-4 SC-271297 1:1000 (Santa Cruz), Mouse anti β-Actin MA5-15739 1:10,000 (invitrogen), Mouse anti alpha-6, produced from hybridoma was a kind gift from Keiji Tanaka. Anti-mouse CD138-APC 561705 1:200 and CD19-PE 115508 1:200 (BD biosciences). Goat anti Mouse 488 A11029 1:400, Goat anti mouse 647 A31571 1:400 (Invitrogen), Goat anti Rabbit 647 AB150075 1:400 (Abcam), Goat anti mouse HRP 115-035-205 1:5000, Goat anti Rabbit HRP | 111-035-003 1:5000 (Jackson labs), K48-TUBE (life-sensors) UM505F 1:50, GFP-GM130 was a kind gift from Martin Lowe, inducible shRNA targeting PSMD6 or luciferase were a kind gift from Yossi Shaul.

### Cell culture and drug treatments

HEK293, A549, HepG2, U2OS, and HeLa cells were originally obtained from ATCC grown in DMEM and used as model systems for cell biology studies including western blot, biochemistry, and imaging. RPMI-8226, U266, and mm1S, originally obtained from ATCC, and 5TGM1, kindly provided by Babatunde Oyajobiwere, were grown in RPMI medium. Cell lines were not authenticated. Cells were routinely tested for mycolplasma contamination. Media were supplemented with 10% fetal bovine serum, 1% Penicillin/streptomycin and l-glutamine (2mmol/l) (Biological industries) at 37 °C with 5% CO_2_.

Concentrations of cell treatments: Tunicamycin (Sigma):5 µg/ml; Monensin (Enco scientific services): 2–4 μM; lithocholylglycine (CAY): 200 μM; MG-132 (Biotest): 20 µM; DBeQ (Sigma-Aldrich): 1 or 10 μM; CB5083 (CAY): 0.5 μM; 3F-NeuAc (400 μM), Nigericin (10 μM), BFA (5 μg/ml). Cell death assessment was done by trypan blue staining and counted with Countess II™ automated cell counter (Thermo Fisher) and by Cell titerGlo assay (Promega). Proliferation of mammalian cells was measured by XTT assay.

### Sucrose cushions and Golgi fractionation

Cells were harvested from four 15 cm plates in cold PBS using a cell scraper and centrifuged at 350 × *g* for 5 min. The supernatant was aspirated, the cells resuspended in 10 ml of swelling buffer (25 mM HEPES pH 7.5, 1.5 mM MgCl_2_, 5 mM KCl, 1 mM DTT, complete protease inhibitor mixture (Roche, Mannheim, Germany), supplemented with energy-mix (20 mM ATP, 150 mM creatine phosphate, 0.1 mM EGTA) and incubated on ice for 10 min. Homogenization was performed using a dounce homogenizer, 20 strokes, on ice. The homogenate was centrifuged at 1000 × *g* for 10 min and the pellet was collected as debris while the supernatant was centrifuged at 100,000 × *g* for 1 h in an SW 41 ultracentrifuge rotor. The supernatant was collected as cytosol and the membranous pellet was resuspended in 1 ml of 0.25 M of sucrose, passing five times through a 25 G syringe. This was overlaid over 4 ml of 0.5 M sucrose and 6 ml 0.86 M of sucrose. This sucrose multi-cushion was centrifuged at 28,000 RPM in an SW41 ultracentrifuge rotor for 1 h. One milliliter fractions were collected from the top using a cut-tip 1 ml pipette. Purity of fractions is validated by SDS-PAGE. Fractions 1–3 were pooled as Golgi-enriched fractions. Fractions 4–10 were pooled as other organelle fractions.

### Immunofluorescence microscopy

A549/HeLa cells, grown on 96-well “cell carrier” plates (Perkin Elmer) were fixed in 4% paraformaldehyde (Electron microscopy sciences) and permeabilized in 0.5% triton (sigma). Cells were blocked in 2% BSA and primary antibodies were introduced for 1 h and secondary antibodies for 30 min, both in 2% BSA. Hoechst staining (Sigma) was done per product protocol. Images were acquired using the “Operetta” high content screening microscope at ×40 magnification and analyzed by “Harmony” software (Perkin Elmer).

For confocal microscopy: A549 cells were permeabilized with digitonin (10 g/ml, 5 min), washed three times with PBS, and fixed in 4% paraformaldehyde and stained as described above. Cells were visualized by VisiScope Confocal Cell Explorer system composed of a Zeiss/Yokogawa spinning disk scanning unit (CSU-W1) coupled with an inverted IX83 microscope (Olympus). Single-focal-plane images were acquired with a 60× oil lens (NA 1.4) and were captured using a PCO-Edge sCMOS camera, controlled by VisiView software (GFP [488 nm], RFP [561 nm], Cy5 [647 nm]) or BFP [405 nm]). Images were reviewed using ImageJ. In all cases, images were enhanced for presentation only. Quantifications were performed on raw image data.

### In vitro ubiquitination assay

Golgi-enriched fractions from sucrose cushions were incubated with energy mix and recombinant HA tagged ubiquitin and either immediately boiled in Laemmli buffer and β-mercaptoethanol or allowed to incubate at room temperature for 30–60 min. All samples were then analyzed by SDS-PAGE using mouse anti HA primary and goat anti-mouse—HRP secondary antibodies. Western blots were quantified using Fiji software.

### Proteasome cleavage reporter assay

Golgi-enriched fractions from drug/siRNA treated HEK293 cells were incubated with suc-LLVY-AMC (Biotest) as per protocol and fluorescence levels were measured over time using a Tecan M200 plate reader (Ex: 360 nm, Em: 460 nm).

### siRNA transfection and RT-PCR analysis

ON-TARGET plus smart-pool siRNAs (Dharmacon) were transfected using lipofectamine 2000 (Invitrogen). mRNA levels were ascertained by real time quantitative PCR using sybr-green (Kapa Biosystems) using the following primers:

Bip TGTTCAACCAATTATCAGCAAACTC

TTCTGCTGTATCCTCTTCACCAGT

CHOP AGAACCAGGAAACGGAAACAGA

TCTCCTTCATGCGCTGCTTT

XBP1s CTGAGTCCGAATCAGGTGCAG

ATCCATGGGGAGATGTTCTGG

PSMD6 AGCCCTAGTAGAGGTTGGCA

AGGAGCCATGTAGGAAGGC,

GAPDH CAACGGATTTGGTCGTATTG

GATGACAAGCTTCCCGTTCT

### Immuno-gold labeling in transmission electron microscopy

HeLa cells were seeded on 3 mm carbon-coated Sapphire disks (Wohlwend GmbH, Switzerland) at a density of 4000 cells/mm^2^ and allowed to settle for 12–18 h. The cells were subsequently fixed by high pressure freezing (HPF) using the Leica EM ICE (Leica Microsystems GmbH, Germany). For HPF, the sapphire disks were removed from the growth medium, and placed between two aluminum planchettes (Wohlwend GmbH, Switzerland) soaked in 1-Hexadecene as cryoprotectant. Freeze substitution and embedding of the HPF-fixed samples were carried out in a temperature-controlled device, AFS2 (Leica Microsystems GmbH, Germany) at − 90 °C for 10 h, using 0.05% (w/v) Uranyl Acetate in dry acetone. The temperature was then raised to −45 °C (5 °C/h) for 5 h followed by three acetone washes. Infiltration with Lowicryl HM20 (Electron Microscopy Sciences, USA) was carried out at increasing concentrations (10%, 25%, for 2 h each). The temperature was then raised to −25 °C (5 °C/h) and infiltration with higher concentrations of Lowicryl HM20 (50%, 75%, 2 h each) was carried out. Finally, 100% Lowicryl HM20 was exchanged three times for every 10 h followed by polymerization under UV light for 48 h. The temperature was increased to 20 °C (5 °C/h) and left under UV light for 12 h.

Thin sections were obtained using an EMUC7 ultramicrotome (Leica microsystems, Vienna, Austria) and were mounted on formvar coated 200 mesh nickel grids. Sections were incubated for 30 min in blocking solution (0.5% gelatin, 0.5% BSA, 0.2% glycine in PBS) and then incubated for 2 h with anti-PSMD6 antibodies (1:20). After washing with PBS containing 0.2% glycine the sections were incubated for 30 min in 10 nm colloidal-gold conjugated goat anti rabbit antibodies (Electron Microscopy Sciences, USA) (1:20) and washed in PBS and bi-distilled water. The immuno-labeled sections were double stained with 2% uranyl acetate and Reynolds lead citrate and observed in a Tecnai T12 Spirit electron microscope (Thermo Fisher Scientific, The Netherlands) operating at 120 kV. Image montages were recorded using an Eagle 2 K × 2 K CCD camera (Thermo Fisher Scientific, The Netherlands) using SerialEM^87^.

### Chemical fixation for ultrastructural analysis

Cells were fixed with 4% paraformaldehyde, 2% glutaraldehyde in 0.1 M cacodylate buffer containing 5 mM CaCl_2_ (pH 7.4), postfixed in 1% osmium tetroxide supplemented with 0.5% potassium hexacyanoferrate tryhidrate and potasssium dichromate in 0.1 M cacodylate for 1H, stained with 2% uranyl acetate in double distilled water for 1H, dehydrated in graded ethanol solutions and embedded in epoxy resin (Agar scientific Ltd., Stansted, UK). Ultrathin sections (70–90 nm) were obtained with an EMUC7 ultramicrotome and were stained with lead citrate and then examined with a Tecnai T12 transmission electron microscope (Thermo Fisher Scientific, The Netherlands). Digital electron micrographs were recorded with a bottom-mounted 4k CMOS camera system (TemCam-F416, TVIPS, Gauting, Germany).

### Immuno-gold staining in scanning electron microscopy

Golgi-enriched fractions collected from sucrose cushions of HEK 293 cells were fixed in 4% paraformaldehyde, 2% glutaraldehyde, in cacodylate buffer containing 5 mM CaCl_2_ pH = 7.4. After fixation, the samples were washed 3–4 times in 0.1 M sodium cacodylate buffer (5 min each) in order to remove all the aldehyde excess. The samples were then plated over-night at 40 °C on silicon wafer coated with poly-l-lysine 1 mg/ml. The samples were then incubated for 1 h in 1% osmium tetroxide in 0.1 M Na cacodylate buffer, washed in cacodylate buffer and then dehydrated in ethanol before being dried in a critical point dryer (CPD) and mounted onto stabs and coated with carbon at 20 nm thickness.

### In-vivo mice work

5Tmm mice, a breed of C57BL/KalwRij mice that are sensitive to mm, were injected with 5TGM1 murine mm cell line and blood levels of IgG2B were measured periodically over 32 days by ELISA. Mice were split into two groups, the control group received 0.35% ethanol in drinking water while the test group received 80 µM monensin (initially solubilized in 70% ethanol) in drinking water. Mice were sacrificed after 5 days of treatment. Spleens and bone marrow were harvested, homogenized, and analyzed by FACS. All mice experiments were approved by the Weizmann Institute’s institutional Animal Care and Use Committee (IACUC) and performed according to IACUC guidelines.

### FACS staining and analysis

Mice were sacrificed, spleens and bones harvested. Bone marrow was flushed out into cold PBS, spleens were processed by passing the spleen through a cell strainer using a syringe plunger. Cells were washed and red blood cells removed using ACK lysis buffer. After washing, cells were stained using PBS−/− containing BSA 0.5% with following antibodies: CD19 (clone 6D5) and CD138 (clone 281-2) for 30 min on ice. After additional washing steps, samples were acquired on a FACSCanto II and analyzed with FlowJo (V10, FlowJo LLC).

### Multispectral imaging flow-cytometry (IFC) analysis

Cells were treated with either LCG (200 μM), monensin (2 μM), Brefeldin A (5 μg/ml), or ethanol as solvent control, for the times indicated in figure legend. Cells were fixed with 1% PFA, permeabilized with 0.1% triton, blocked in 2% BSA in PBS and stained with anti-Giantin antibody (ab80864) for human cells, anti-GRASP65 antibody (ab30315) for mouse cells. 5TGM1 cells were also stained with K48-TUBE (life sensors). Cells were then incubated with Alexa Fluor® 568-conjugated secondary antibodies (Biolegend) and Hoechst (Sigma Aldrich). Cells were imaged using multispectral imaging flow cytometry (ImageStreamX mark II imaging flow-cytometer; Amnis Corp, Seattle, WA, Part of Luminex). Approximately 5 × 10^4^ cells were collected from each sample and data were analyzed using the manufacturer’s image analysis software (IDEAS 6.2; Amnis Corp). Images were compensated for fluorescent dye overlap by using single-stain controls. Cells were gated for single cells, using the area and aspect ratio features, and for focused cells, using the Gradient RMS feature^88^. For evaluation of Golgi integrity two features were calculated based on the Golgi staining: Minor Axis Intensity (the intensity weighted narrowest dimension of the ellipse of best fit) and Area (the number of microns squared within a mask). These were calculated on the Threshold_60 mask that includes the 60% highest intensity pixels of the Golgi staining (The Area_Threshold_60 was also considered as the Golgi area). These two features were plotted as a bivariate plot and the different Golgi morphologies were gated according to visual inspection and control samples. For quantification of apoptotic cells, single, focused cells were plotted for the contrast of the bright field channel vs. the area of the 50% highest intensity pixels of the DAPI staining (defined by the Threshold_50 mask). Cells with high contrast and low area (condensed) of the DNA staining were considered apoptotic. Accumulation of K48 in the Golgi was calculated by measuring the intensity of the K48 staining within the Golgi mask, using the morphology mask on the GRASP65 staining.

### SPECS method for determination of sialylation

Cells were incubated in the presence of 1 μmol ManNAz for 5 h. Conditioned medium was collected and 500 nM of Sulfo-DBCO-biotin was added to facilitate biotinylation of azide-labeled glycoproteins. Non-reacted Sulfo-DBCO-biotin was removed on VivaSpin20 columns. Biotinylated glycoproteins were detected by WB using Avidin-HRP.

### LCG washout

HeLa cells were transfected with GalT-YFP expression vectors. Cells were sorted to select average intensity cells and seeded on fibronectin-coated plates. Cells were treated with LCG (200 µM) or EtOH as vehicle control for six hours and then washed three times with DMEM medium. Images were taken in 20 min intervals, using Hermes WiScan instrument.

### Viability assays by cell titer-Glo

RPMI-8226 cells were infected with either expression vectors for GFP or GFP-GM130, or uninfected, and treated with LCG (200 µM) or ethanol as vehicle control for 15 h. Cells were then assayed using Cell titer-Glo as specified by the manufacturer.

## Supplementary information

Supplementary Information

Description of Additional Supplementary Files

Supplementary Data 1

## Data Availability

The source data underlying Figs. 1H, 2G, 3F, 4B are provided as a Source Data file. Further data available on request from the authors.

## Source data

Source Data
