# Supplementary material for: Golgi organization is regulated by proteasomal degradation
Source: Nat Commun. 2020 Jan 21;11:409. doi: 10.1038/s41467-019-14038-9 (PMC6972958; doi:10.1038/s41467-019-14038-9)
Supplement: Supplementary file 1 — Supplementary Information [file 41467_2019_14038_MOESM1_ESM.pdf]

Supplementary figures

Fine-tuning regulation of Golgi organization is mediated by proteasomal degradation

Eisenberg-Lerner at al.

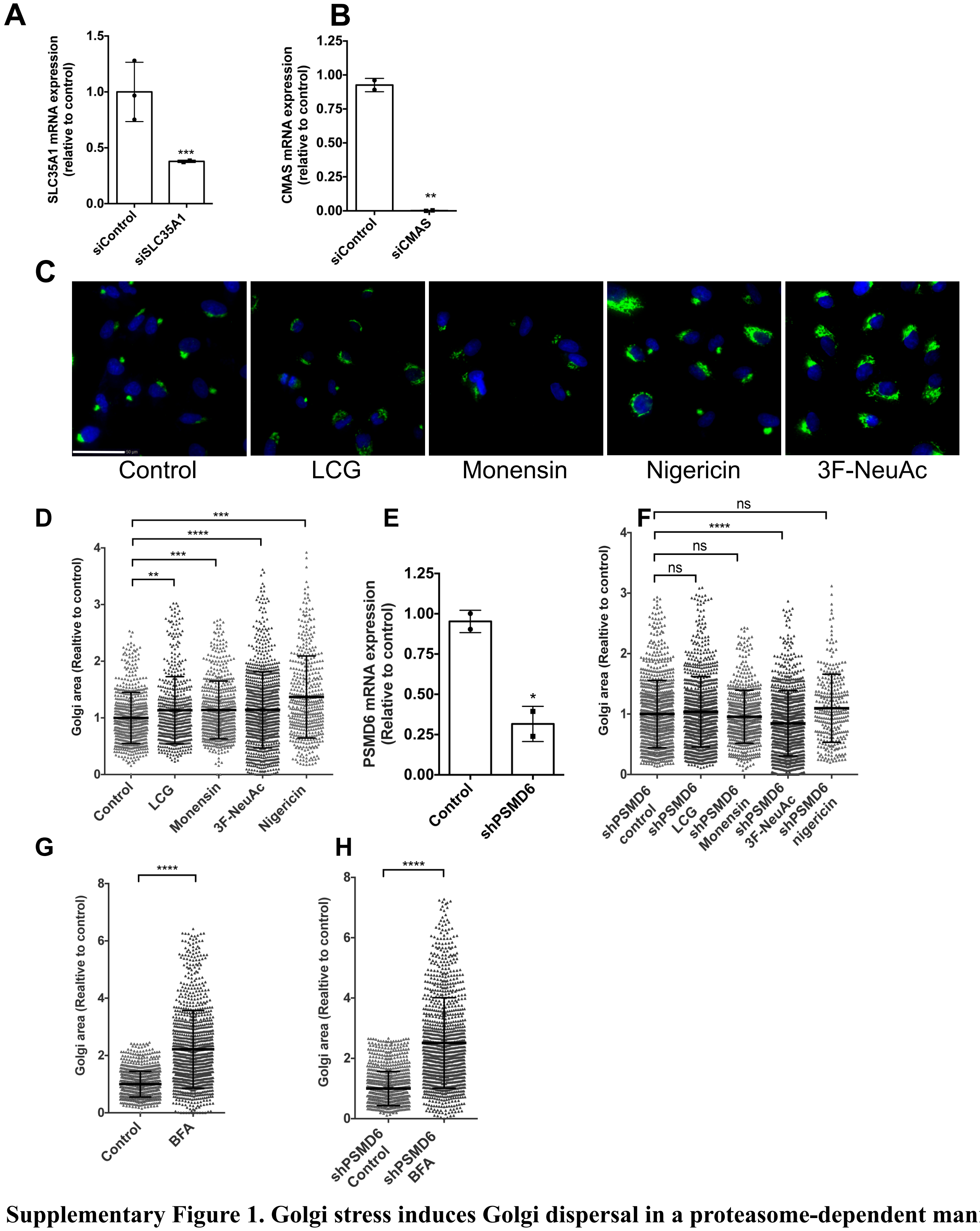

Supplementary Figure 1. Golgi stress induces Golgi dispersal in a proteasome-dependent manner.

(A,B) A549 cells stably expressing an inducible shRNA for PSMD6 were transfected with siRNA targeting wither SLC35A1, CMAS, or non-targeting control. 72 hours post transfection, mRNA levels of SLC35A1 (A) or CMAS (B) were determined by qPCR. n=3 independent experiments. Error bars=SD. p(SLC35A1)=0.0008; p(CMAS)= 0.0014; (two tailed unpaired t-test). (C) Immunofluorescence images of A549 cells treated with either LCG (200μM), monensin (2μM), 3F-NeuAc (400μM), Nigericin (10 μM) for six hours. Images are representative of 600 cells per condition. Giantin (green); Nuclei (blue). Scale bar = 50μM. (D) Single cell quantification of the Golgi area of cells treated as in C. n=5070 cells. Error bars=SD. p(LCG=0.0043; p(mon)=0.0027; p(3F-NeuAc)=0.0005; p(nigericin)<0.0001 (one way ANOVA with Holm-Sidak's multiple comparisons test). (E) A549 cells expressing an inducible knock down of PSMD6 were stimulated with Doxycycline to induce shRNA expression for 72 hours. PSMD6 mRNA levels were determined by qPCR. n=3 biologically independent samples. p=0.02 (two tailed unpaired t-test). (F) Following 72 hours of Doxycycline treatment, cells were stimulated as indicated for six hours and Golgi area was quantified in single cells. n=5335 cells. Error bars=SD. ns: non-significant.; ****p<0.0001 (Bonferroni multiple comparisons one way ANOVA test). (G, H) A549 cells expressing an inducible knock down of PSMD6 were treated with BFA (5 μg/ml) either with or without stimulation with Doxycycline to induce PSMD6 knockdown. The Golgi area was quantified in single cells under these conditions. N=1070 cells. p(BFA)<0.0001.

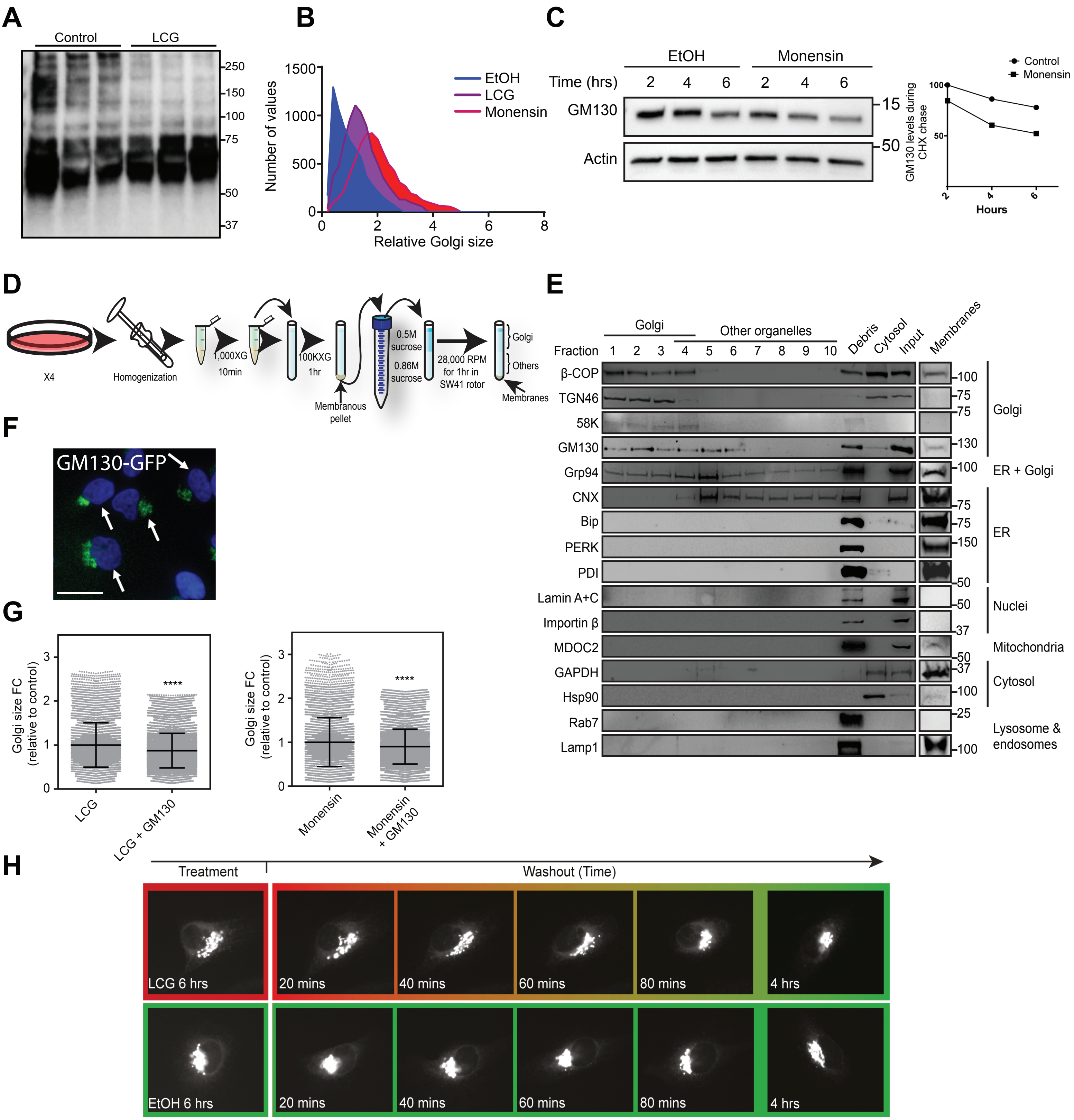

Supplementary Figure 2. Degradation of GM130 mediates reversible Golgi dispersal under stress. (A) A549 cells were incubated for 6 hours with LCG or EtOH in serum-free media. Sialylated proteins were detected in the secretome using Avidin-HRP WB. (B) The Frequency distribution of the relative Golgi size of A549 cells that were treated with LCG (200µM) for 6 hours compared to cells treated with EtOH as control based on GRASP65 staining. Error bars=SD. n= 3 independent experiments, 6790 cells were/condition on average. (C) RPMI-8226 cells were treated with cycloheximide (CHX 100µg/ml) and either Monensin (4 µM) or EtOH for the indicated times, and analyzed by WB with the indicated antibodies. n=3 different experiments. Graph shows quantification. (D) Schematic representation of cellular fractionation on sucrose cushions. (E) HEK293 cells were fractionated on sucrose cushions and the fractions were separated on SDS-PAGE and analyzed with the indicated antibodies representing different cellular organelles. n=3 different experiments. (F) A549 cells were transfected with GFP-GM130. Shown are representative cells images; White arrows point to cells expressing GFP-GM130. Scale bar = 20µM. (G) Cells transfected with GFP-GM130 or GFP as control were treated with either LCG (left) or Monensin (right) for 6 hours. The relative Golgi size of GM130 expressing cells, compared to GFP expressing cells, was determined based on Giantin staining. To calculate relative Golgi area, each data point was divided by the Golgi area mean of treatment alone. n>4000 cells. Error bars=SD. $p<0.0001$ (unpaired two tailed t-test). (H) HeLa cells expressing GalT-YFP were treated with LCG (200 µM) or EtOH as control for six hours, then washed three times with medium and imaged in 20 minutes intervals for four hours. Representative images of 50 cells are presented.

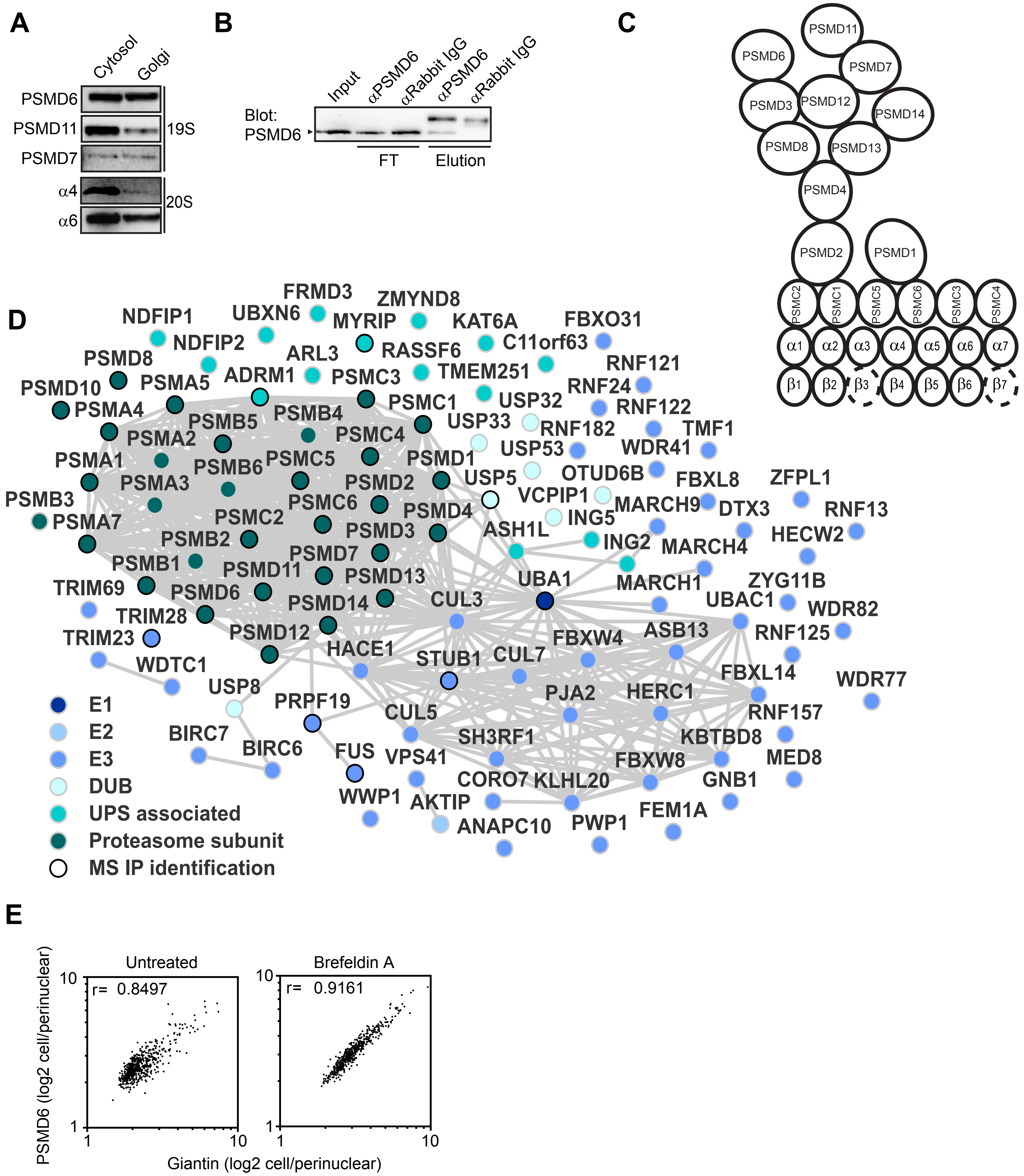

Supplementary Figure 3. Full proteasomes are associated with Golgi membranes. (A) Cytosolic and Golgi-enriched fractions from sucrose cushions (see methods for details) were analyzed by WB analysis for the indicated 19S and 20S proteasome subunits. Image is representative of three different experiments. (B-D) PSMD6 was immunoprecipitated from Golgi fractions following solubilization with DDM (1%). Anti rabbit IgG was used as mock control. Input: Golgi fraction; FT: flow-through of the reaction; Elution: extraction of the precipitated proteins from the immunocomplex. n=2. (C) Schematic representation of the full proteasome subunits. Full lines: subunits identified by mass-spectrometry in interaction with PSMD6 from Golgi fractions. Dashed: two subunits of the full proteasome that were not identified in the analysis. (D) Interaction map of ubiquitin-proteasome system related proteins detected by pull down of either Ubiquitin Interacting Motif (UIM)-GST or PSMD6 from purified Golgi fractions and mass spectrometry analysis. (E) The localization of PSMD6 and Giantin was determined in HeLa cells treated with Brefeldin A where indicated by immunofluorescence. The correlation between the ratios of total cell/perinuclear distributions of the localization of PSMD6 and Giantin was analyzed.

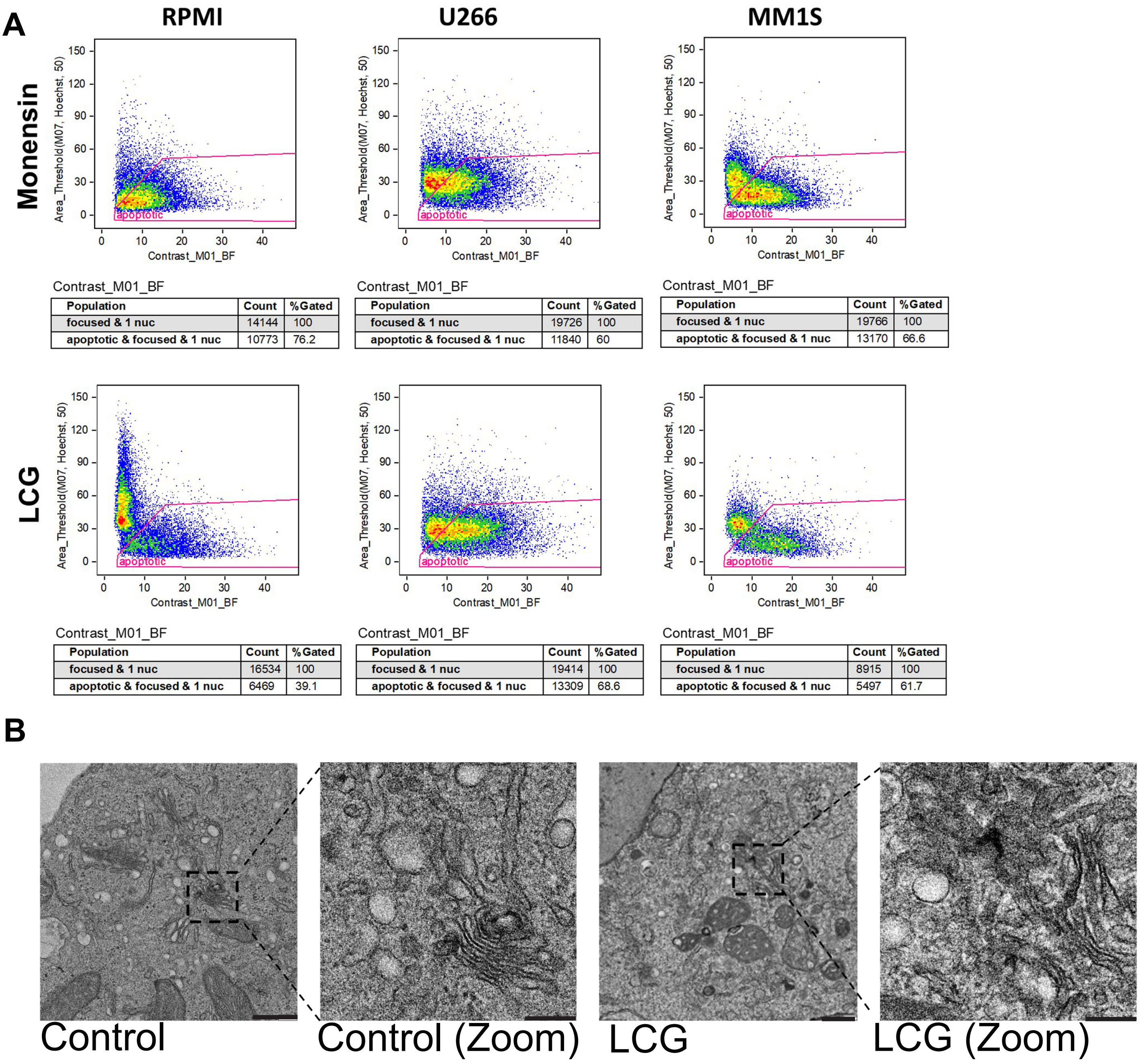

Supplementary Figure 4. Golgi stress induces cell death and Golgi dispersal in Multiple myeloma cells. (A) The indicated cell lines were treated with either LCG (200μM), monensin (2μM), or control for 6 hours and imaged by Imaging Flow Cytometer ImageStreamX mark II for nuclei by Hoechst morphology to identify apoptotic cells. Shown is the single cell distribution of >25,000 cells. (B) RPMI-8226 cells were treated with LCG (200μM) or control for 6 hours and imaged by transmission electron microscopy. Scale bar= 500nM; Scale bar in zoom images= 200nM.

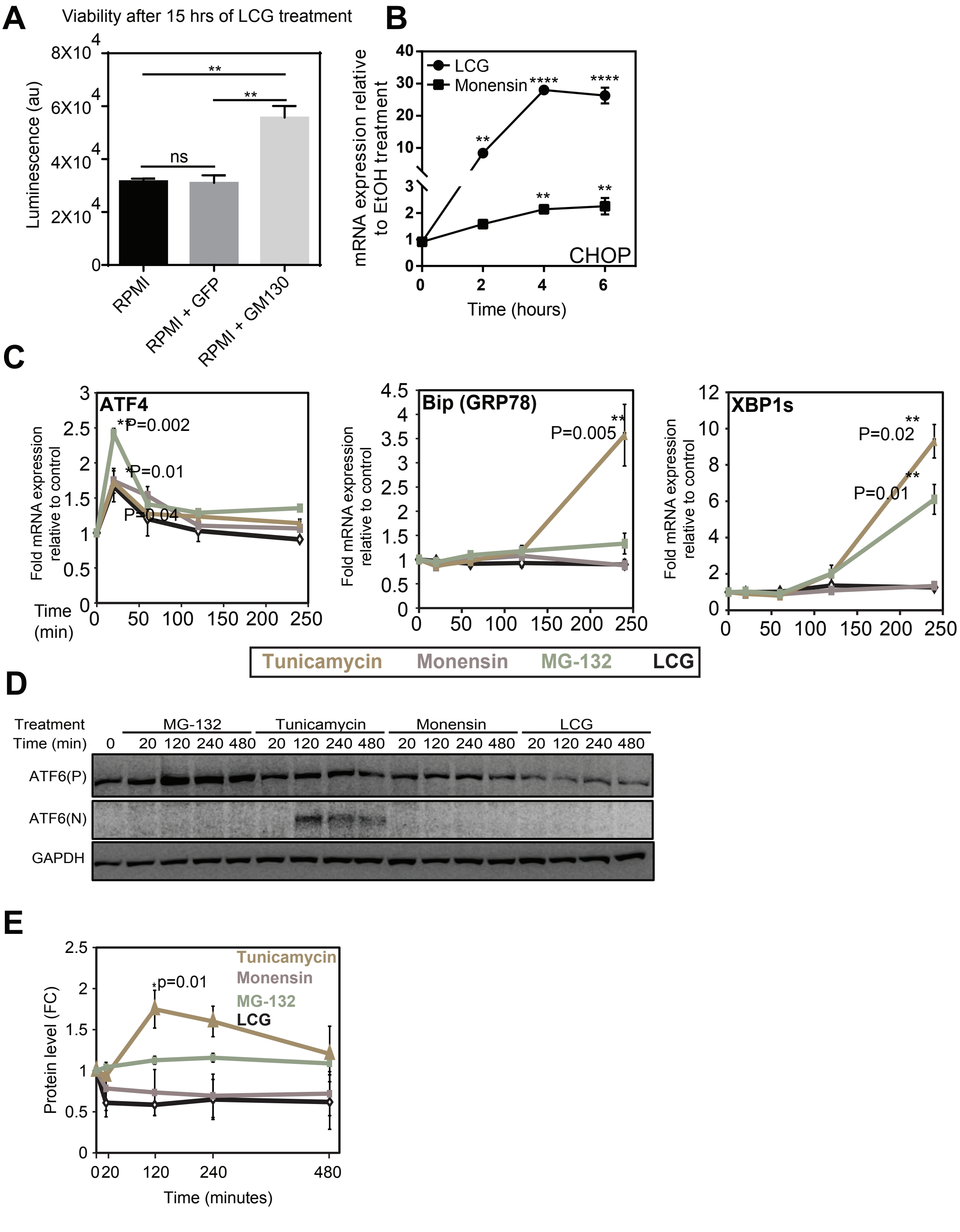

Supplemetary Figure 5. Golgi stress is independent from cannonical UPR.
(A) RPMI-8226 cells over-expressing either GFP or GFP-GM130, or non-transfected cells, were treated with LCG (200µM) or EtOH as control for 15 hours, and subjected to Cell titer-Glo viability assay. Error bars=SEM. n=2 independent experiments. p(RPMI vs RPMI-GM130)=0.0036; p(RPMI-GFP vs RPMI-GM130)=0.0032 (one way ANOVA with Tukey's multiple comparisons test). (B) CHOP mRNA levels were measured by qPCR from RPMI-8226 cells treated with LCG (200µM). n=3 independent experiments. **p<0.01; ****p<0.0001. (C) qPCR quantification of mean ATF4, Bip (Grp78), and spliced XBP1, mRNA levels ± SEM in HEK293 cells that were treated as indicated. n=3 independent experiments. (D) Western blot analysis of ATF6 cleavage in cells treated as indicated, showing full size ATF6 [ATF6(P)] and cleaved, nuclear, AT6 [ATF6(N)]. (E) Quantification mean ATF6 cleavage ± SEM in cells treated as indicated (n=3 independent experiments), normalized to GAPDH levels, showing a peak in ATF6 cleavage after 120 minutes of tunicamycin treatment p=0.01

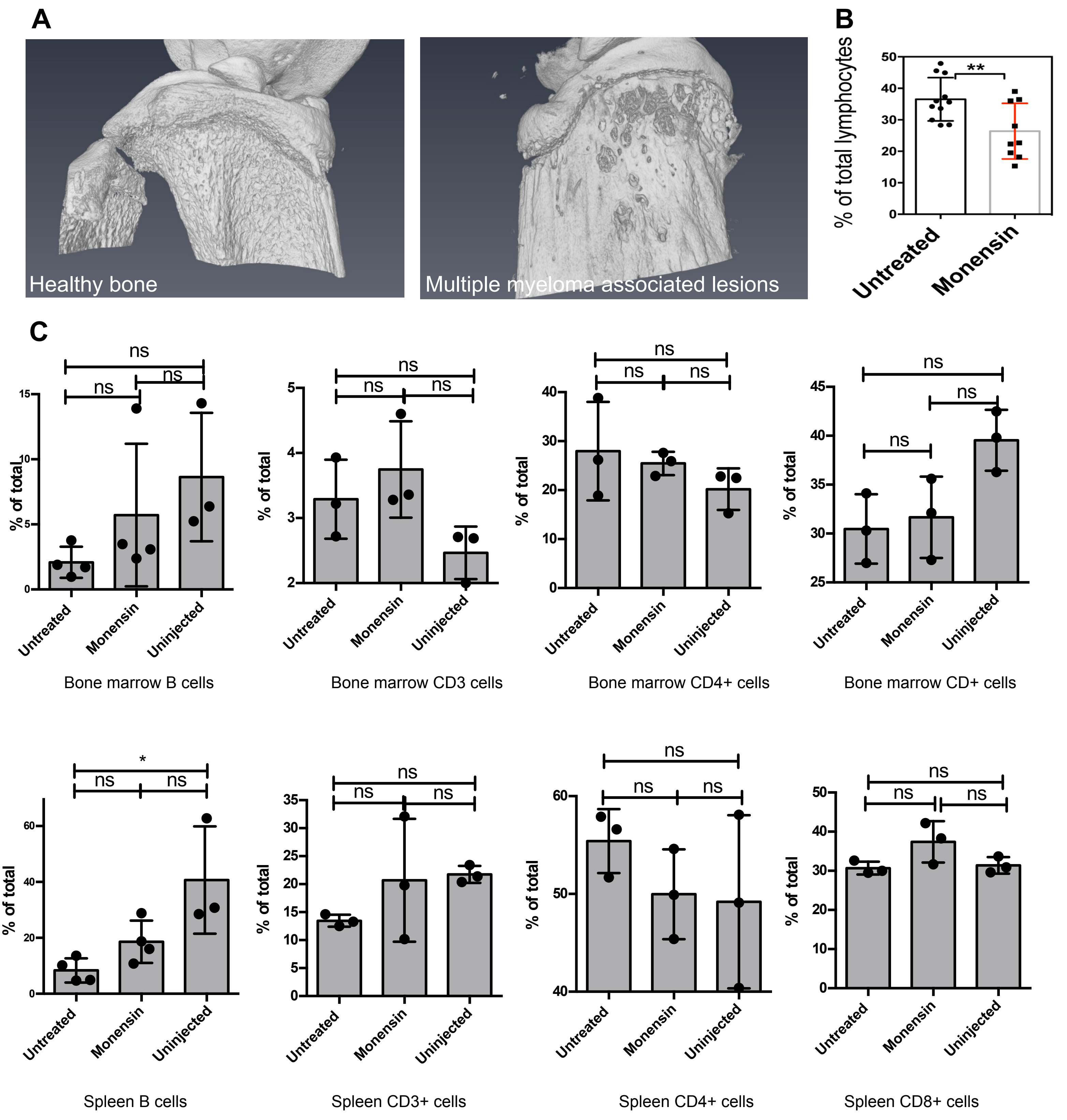

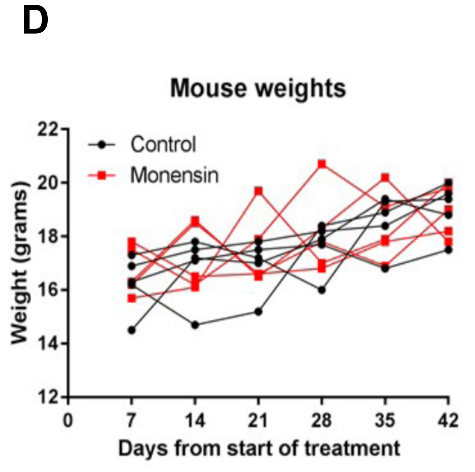

Supplemetary Figure 6. Monensin is toxic to mm cells.
(A) Examples of bone marrow lesions as observed by Ex vivo CT. (B) FACS analysis and quantification of multiple myeloma cells in bone marrow of control vs. monensin-treated mice n=20 mice in three independent experiments. Values were normalized to all-mean value. p =0.0099 (unpaired t-test). (C) FACS analysis quantification of the indicated cell types in bone marrows and spleens of control vs. monensin-treated mm mice. n=9 mice. Error bars: SEM. N: non-significant (one way ANOVA with Tukey's multiple comparisons test). (D) Body weight of C57BL6/KaLwRij mice administered with monensin (80 μM) in the drinking water over 42 days of treatment.
