# Supplementary material for: Golgi organization is regulated by proteasomal degradation
Source: Nat Commun. 2020 Jan 21;11:409. doi: 10.1038/s41467-019-14038-9 (PMC6972958; doi:10.1038/s41467-019-14038-9)
Supplement: Supplementary file 2 — Description of Additional Supplementary Files [file 41467_2019_14038_MOESM2_ESM.pdf]

Description of Additional Supplementary Files

File Name: Supplementary Data 1
Description: Proteasomal peptides identified by MS analysis.
