# Supplementary material for: Golgi organization is regulated by proteasomal degradation
Source: Nat Commun. 2020 Jan 21;11:409. doi: 10.1038/s41467-019-14038-9 (PMC6972958; doi:10.1038/s41467-019-14038-9)
Supplement: Supplementary file 3 — Supplementary Data 1 [file 41467_2019_14038_MOESM3_ESM.pdf]

Table 1. Proteasomal peptides identified by MS analysis.

Mass spectrometry analysis of proteins that co-precipitated with PSMD6 from Golgi fractions following solubilization with DDM (1%) and that were significantly higher than in mock antibody immunoprecipitation.

Sequence	Gene names	Protein names
AQPAQPADEPAEKADEPMEH	PSMA1	Proteasome subunit alpha type-1
ETLPAEQDLTTK	PSMA1	Proteasome subunit alpha type-1
IHQIEYAMEAVK	PSMA1	Proteasome subunit alpha type-1
LVSLIGSK	PSMA1	Proteasome subunit alpha type-1
NQYDNDVTVWSPQGR	PSMA1	Proteasome subunit alpha type-1
NVSIGIVGK	PSMA1	Proteasome subunit alpha type-1
AANGVVLATEKK	PSMA2	Proteasome subunit alpha type-2
SILYDER	PSMA2	Proteasome subunit alpha type-2
SVHKVEPITK	PSMA2	Proteasome subunit alpha type-2
LFNVDR	PSMA3	Proteasome subunit alpha type-3
VFQVEYAMK	PSMA3	Proteasome subunit alpha type-3
EVEQLIK	PSMA4	Proteasome subunit alpha type-4
LSAEKVEIATLTR	PSMA4	Proteasome subunit alpha type-4
QKEVEQLIK	PSMA4	Proteasome subunit alpha type-4
SALALAIK	PSMA4	Proteasome subunit alpha type-4
TTIFSPEGR	PSMA4	Proteasome subunit alpha type-4
VEIATLTR	PSMA4	Proteasome subunit alpha type-4
AIGSASEGAQSSLQEVYHK	PSMA5	Proteasome subunit alpha type-5
ITSPLMEPSSIEK	PSMA5	Proteasome subunit alpha type-5
SSLIILK	PSMA5	Proteasome subunit alpha type-5
AINQGGLTSVAVR	PSMA6	Proteasome subunit alpha type-6
HITIFSPEGR	PSMA6	Proteasome subunit alpha type-6
KVPDKLLDSSTVTHLFK	PSMA6	Proteasome subunit alpha type-6
LLDSSTVTHLFK	PSMA6	Proteasome subunit alpha type-6
QTESTSFLEK	PSMA6	Proteasome subunit alpha type-6
GRDIVVLGVEK	PSMA7	Proteasome subunit alpha type-7
ILNPEEIEK	PSMA7	Proteasome subunit alpha type-7
NIELAVMR	PSMA7	Proteasome subunit alpha type-7
NYTDEAIETDDLTIK	PSMA7	Proteasome subunit alpha type-7
RPFGISALIVGFDFDGTPR	PSMA7	Proteasome subunit alpha type-7
ALLEVVQSGGK	PSMA7;PSMA8	Proteasome subunit alpha type-7
LYQTDPSGTYHAWK	PSMA7;PSMA8	Proteasome subunit alpha type-7
AGGSASAMLQPLLDNQVGFK	PSMB1	Proteasome subunit beta type-1
AMTTGAIAAMLSTILYSR	PSMB1	Proteasome subunit beta type-1

DVFISAAER	PSMB1	Proteasome subunit beta type-1
EGIREETVSLR	PSMB1	Proteasome subunit beta type-1
LSEGFSIHTR	PSMB1	Proteasome subunit beta type-1
NMQNVEHVPLSLDR	PSMB1	Proteasome subunit beta type-1
AVELLR	PSMB2	Proteasome subunit beta type-2
MEYLIGIQGPDYVLVASDR	PSMB2	Proteasome subunit beta type-2
NGYELSPTAAANFTR	PSMB2	Proteasome subunit beta type-2
YYTPTISR	PSMB2	Proteasome subunit beta type-2
GVEIEGPLSTETNWDIAHMISGFE	PSMB4	Proteasome subunit beta type-4
AIYQATYR	PSMB5	Proteasome subunit beta type-5
ATAGAYIASQTVK	PSMB5	Proteasome subunit beta type-5
ATAGAYIASQTVKK	PSMB5	Proteasome subunit beta type-5
DAYSGGAVNLYHVR	PSMB5	Proteasome subunit beta type-5
ISVAAASK	PSMB5	Proteasome subunit beta type-5
RAIYQATYR	PSMB5	Proteasome subunit beta type-5
VSSDNVADLHEK	PSMB5	Proteasome subunit beta type-5
LAAIAESGVER	PSMB6	Proteasome subunit beta type-6
QVLLGDQIPK	PSMB6	Proteasome subunit beta type-6
TTTGSYIANR	PSMB6	Proteasome subunit beta type-6
VTDKLTPIHDR	PSMB6	Proteasome subunit beta type-6
AICTEAGLMALR	PSMC1	26S protease regulatory subunit 4
APQETYADIGGLDNQIQEIK	PSMC1	26S protease regulatory subunit 4
AVANQTSATFLR	PSMC1	26S protease regulatory subunit 4
ESVELPLTHPEYYEEMGIKPPK	PSMC1	26S protease regulatory subunit 4
GPDAASKLPLVTPHTQCR	PSMC1	26S protease regulatory subunit 4
IEFPLPDEK	PSMC1	26S protease regulatory subunit 4
IETLDPALIRPGR	PSMC1	26S protease regulatory subunit 4
IETLDPALIRPGRIDR	PSMC1	26S protease regulatory subunit 4
IFQIHTSR	PSMC1	26S protease regulatory subunit 4
IKDYLLMEEEFIR	PSMC1	26S protease regulatory subunit 4
KIEFPLPDEK	PSMC1	26S protease regulatory subunit 4
KKYEPPVPTR	PSMC1	26S protease regulatory subunit 4
KQEGTPEGLYL	PSMC1	26S protease regulatory subunit 4
KYEPPVPTR	PSMC1	26S protease regulatory subunit 4
LPLVTPHTQCR	PSMC1	26S protease regulatory subunit 4
NQEQMKPLEEKQEEER	PSMC1	26S protease regulatory subunit 4
TKGPDAASKLPLVTPHTQCR	PSMC1	26S protease regulatory subunit 4
TMLELLNQLDGFDSR	PSMC1	26S protease regulatory subunit 4
VAEEHAPSIVFIDEIDAIGTK	PSMC1	26S protease regulatory subunit 4
VAEEHAPSIVFIDEIDAIGTKR	PSMC1	26S protease regulatory subunit 4
VHAVIGVLMDDTDPLVTVMK	PSMC1	26S protease regulatory subunit 4

VVGSELIQK	PSMC1	26S protease regulatory subunit 4
ACLIFFDEIDAIGGAR	PSMC2	26S protease regulatory subunit 7
ALDEGDIALLK	PSMC2	26S protease regulatory subunit 7
DFLEAVNK	PSMC2	26S protease regulatory subunit 7
DIRFELLAR	PSMC2	26S protease regulatory subunit 7
ELFEMAR	PSMC2	26S protease regulatory subunit 7
EVVETPLLHPER	PSMC2	26S protease regulatory subunit 7
FDDGAGGDNEVQR	PSMC2	26S protease regulatory subunit 7
FELLAR	PSMC2	26S protease regulatory subunit 7
FSATPR	PSMC2	26S protease regulatory subunit 7
FVNLGIEPPK	PSMC2	26S protease regulatory subunit 7
FVNLGIEPPKGVLLFGPPGTGK	PSMC2	26S protease regulatory subunit 7
FVVDLSDQVAPTDIEEGMR	PSMC2	26S protease regulatory subunit 7
GVLLFGPPGTGK	PSMC2	26S protease regulatory subunit 7
IATEKDFLEAVNK	PSMC2	26S protease regulatory subunit 7
IDPTVTMMQVEEKPDVTYSDVGGCK	PSMC2	26S protease regulatory subunit 7
IDPTVTMMQVEEKPDVTYSDVGGCKEQIEK	PSMC2	26S protease regulatory subunit 7
IEFSLPDLEGR	PSMC2	26S protease regulatory subunit 7
IINADSEDPKYIINVK	PSMC2	26S protease regulatory subunit 7
INELTGIK	PSMC2	26S protease regulatory subunit 7
INELTGIKESDTGLAPPALWDLAADKQTLQSEQPLQVA R	PSMC2	26S protease regulatory subunit 7
KACLIFFDEIDAIGGAR	PSMC2	26S protease regulatory subunit 7
KIEFSLPDLEGR	PSMC2	26S protease regulatory subunit 7
KINELTGIK	PSMC2	26S protease regulatory subunit 7
LCPNSTGAEIR	PSMC2	26S protease regulatory subunit 7
LREVVETPLLHPER	PSMC2	26S protease regulatory subunit 7
NKYQIHIPLPPK	PSMC2	26S protease regulatory subunit 7
PDYLGADQRK	PSMC2	26S protease regulatory subunit 7
QTLQSEQPLQVAR	PSMC2	26S protease regulatory subunit 7
QVEDDIQQLLK	PSMC2	26S protease regulatory subunit 7
QVEDDIQQLLKK	PSMC2	26S protease regulatory subunit 7
SVCTEAGMFAIR	PSMC2	26S protease regulatory subunit 7
TDACFIR	PSMC2	26S protease regulatory subunit 7
TMLELINQLDGFDPR	PSMC2	26S protease regulatory subunit 7
TYGQSTYSR	PSMC2	26S protease regulatory subunit 7
VLMATNR	PSMC2	26S protease regulatory subunit 7
VLMATNRPDTLDPALMRPGR	PSMC2	26S protease regulatory subunit 7
YIINVK	PSMC2	26S protease regulatory subunit 7
YQIHIPLPPK	PSMC2	26S protease regulatory subunit 7
AMEVDERPTEQYSDIGGLDK	PSMC3	26S protease regulatory subunit 6A
AMEVDERPTEQYSDIGGLDKQIQELVEAIVLPMNHK	PSMC3	26S protease regulatory subunit 6A

ATVWDEAEQDGIGEEVLK	PSMC3	
AVCVEAGMIALR	PSMC3	26S protease regulatory subunit 6A
AVCVEAGMIALRR	PSMC3	26S protease regulatory subunit 6A
CTDDFNGAQCK	PSMC3	26S protease regulatory subunit 6A
EKAPSIIFIDELDAIGTK	PSMC3	26S protease regulatory subunit 6A
EKFENLGIQPPK	PSMC3	26S protease regulatory subunit 6A
GATELTHEDYMEGILEVQAK	PSMC3	26S protease regulatory subunit 6A
GVLMYGPPGTGK	PSMC3	26S protease regulatory subunit 6A
IMQIHSR	PSMC3	26S protease regulatory subunit 6A
KIEFPMPNEEAR	PSMC3	26S protease regulatory subunit 6A
KMNVSPDVNYEELAR	PSMC3	26S protease regulatory subunit 6A
LKPGDLVGVNK	PSMC3	26S protease regulatory subunit 6A
LLDSEIK	PSMC3	26S protease regulatory subunit 6A
MNVSPDVNYEELAR	PSMC3	26S protease regulatory subunit 6A
MSTEEIIQR	PSMC3	26S protease regulatory subunit 6A
QIQELVEAIVLPMNHK	PSMC3	26S protease regulatory subunit 6A
QTYFLPVIGLVDAEK	PSMC3	26S protease regulatory subunit 6A
TMLELLNQLDGFQPNTQVK	PSMC3	26S protease regulatory subunit 6A
TRLLDSEIK	PSMC3	26S protease regulatory subunit 6A
VDILDPALLR	PSMC3	26S protease regulatory subunit 6A
VIAATNRVDILDPALLR	PSMC3	26S protease regulatory subunit 6A
VTHELQAMK	PSMC3	26S protease regulatory subunit 6A
VTHELQAMKDK	PSMC3	26S protease regulatory subunit 6A
ADTLDPALLRPGR	PSMC4	26S protease regulatory subunit 6B
ADTLDPALLRPGRLDR	PSMC4	26S protease regulatory subunit 6B
AQDEIPALSVSRPQTGLSFLGPEPEDLEDLYSR	PSMC4	26S protease regulatory subunit 6B
AVAHHTTAAFIR	PSMC4	26S protease regulatory subunit 6B
EAVELPLTHFELYK	PSMC4	26S protease regulatory subunit 6B
EFLHAQEEVKR	PSMC4	26S protease regulatory subunit 6B
ELLKPNASVALHK	PSMC4	26S protease regulatory subunit 6B
ENAPAIIFIDEIDAIATK	PSMC4	26S protease regulatory subunit 6B
ENAPAIIFIDEIDAIATKR	PSMC4	26S protease regulatory subunit 6B
FDAQTGADREVQR	PSMC4	26S protease regulatory subunit 6B
GVLMYGPPGCGK	PSMC4	26S protease regulatory subunit 6B
IEFPLPDRR	PSMC4	26S protease regulatory subunit 6B
ILLELLNQMDGFDQNVNVK	PSMC4	26S protease regulatory subunit 6B
ILSTIDR	PSMC4	26S protease regulatory subunit 6B
ILSTIDRELLKPNASVALHK	PSMC4	26S protease regulatory subunit 6B
IQSIPLVIGQFLEAVDQNTAIVGSTTGSNYYVR	PSMC4	26S protease regulatory subunit 6B
ISGADINSICQESGMLAVR	PSMC4	26S protease regulatory subunit 6B
KEFLHAQEEVKR	PSMC4	26S protease regulatory subunit 6B

KIEFPLPDRR	PSMC4	26S protease regulatory subunit 6B
LIFSTITSK	PSMC4	26S protease regulatory subunit 6B
LQQELEFLEVQEEYIKDEQK	PSMC4	26S protease regulatory subunit 6B
MEEIGILVEK	PSMC4	26S protease regulatory subunit 6B
MNLSEEVDLEDYVARPDK	PSMC4	26S protease regulatory subunit 6B
RFDAQTGADREVQR	PSMC4	26S protease regulatory subunit 6B
TVIKKDEQEHEFYK	PSMC4	26S protease regulatory subunit 6B
VVGSEFVQK	PSMC4	26S protease regulatory subunit 6B
YLGEGPR	PSMC4	26S protease regulatory subunit 6B
ALDGPEQMELEEGK	PSMC5	26S protease regulatory subunit 8
ALDGPEQMELEEGKAGSGLR	PSMC5	26S protease regulatory subunit 8
AVAHHTDCTFIR	PSMC5	26S protease regulatory subunit 8
EELQLLQEQGSYVGEVVR	PSMC5	26S protease regulatory subunit 8
EHAPSIIFMDEIDSIGSSR	PSMC5	26S protease regulatory subunit 8
ELFVMAR	PSMC5	26S protease regulatory subunit 8
EVIELPVKHPELFEALGIAQPK	PSMC5	26S protease regulatory subunit 8
FIGEGAR	PSMC5	26S protease regulatory subunit 8
FVVDVDKNIDINDVTPNCR	PSMC5	26S protease regulatory subunit 8
GVCTEAGMYALR	PSMC5	26S protease regulatory subunit 8
IAELMPGASGAEVK	PSMC5	26S protease regulatory subunit 8
IDILDSALLRPGR	PSMC5	26S protease regulatory subunit 8
IDILDSALLRPGRIDR	PSMC5	26S protease regulatory subunit 8
IEELQLIVNDK	PSMC5	26S protease regulatory subunit 8
ILPNKVDPLVSLMMVEK	PSMC5	26S protease regulatory subunit 8
KIAELMPGASGAEVK	PSMC5	26S protease regulatory subunit 8
KIEFPPPNEEAR	PSMC5	26S protease regulatory subunit 8
LEGGSGGDSEVQR	PSMC5	26S protease regulatory subunit 8
LLREELQLLQEQGSYVGEVVR	PSMC5	26S protease regulatory subunit 8
NDSYTLHK	PSMC5	26S protease regulatory subunit 8
QYYLSK	PSMC5	26S protease regulatory subunit 8
RVHVTQEDFEMAVAK	PSMC5	26S protease regulatory subunit 8
TMLELLNQLDGFEATK	PSMC5	26S protease regulatory subunit 8
VALRNDSYTLHK	PSMC5	26S protease regulatory subunit 8
VHVTQEDFEMAVAK	PSMC5	26S protease regulatory subunit 8
VPDSTYEMIGGLDK	PSMC5	26S protease regulatory subunit 8
GVLLYGPPGTGK	PSMC5;SPATA5	26S protease regulatory subunit 8
ADHDFVVQEDFMK	PSMC6	26S protease regulatory subunit 10B
ALQDYR	PSMC6	26S protease regulatory subunit 10B
ALQSVGQIVGEVLK	PSMC6	26S protease regulatory subunit 10B
AVASQLDCNFLK	PSMC6	26S protease regulatory subunit 10B
DHQPCIIFMDEIDAIGGR	PSMC6	26S protease regulatory subunit 10B

DHQPCIIFMDEIDAIGGRR	PSMC6	26S protease regulatory subunit 10B
DKALQDYR	PSMC6	26S protease regulatory subunit 10B
EMFNYAR	PSMC6	26S protease regulatory subunit 10B
EQLKELTK	PSMC6	26S protease regulatory subunit 10B
EVDPLVYNMSHEDPGNVSYSEIGGLSEQIR	PSMC6	26S protease regulatory subunit 10B
EVIELPLTNPELFQR	PSMC6	26S protease regulatory subunit 10B
FSEGTSADREIQR	PSMC6	26S protease regulatory subunit 10B
GCLLYGPPGTGK	PSMC6	26S protease regulatory subunit 10B
HGEIDYEAIVK	PSMC6	26S protease regulatory subunit 10B
IHAGPITK	PSMC6	26S protease regulatory subunit 10B
IHIDLPNEQAR	PSMC6	26S protease regulatory subunit 10B
KIHIDLPNEQAR	PSMC6	26S protease regulatory subunit 10B
LLEHKEIDGR	PSMC6	26S protease regulatory subunit 10B
LSDGFNGADLR	PSMC6	26S protease regulatory subunit 10B
MIMATNRPDTLDPALLRPGR	PSMC6	26S protease regulatory subunit 10B
NVCTEAGMFAIR	PSMC6	26S protease regulatory subunit 10B
RFSEGTSADREIQR	PSMC6	26S protease regulatory subunit 10B
TLMELLNQMDGFDTLHR	PSMC6	26S protease regulatory subunit 10B
VALDMTTLTIMR	PSMC6	26S protease regulatory subunit 10B
VVSSSIVDK	PSMC6	26S protease regulatory subunit 10B
VVSSSIVDKYIGESAR	PSMC6	26S protease regulatory subunit 10B
YVVGCR	PSMC6	26S protease regulatory subunit 10B
AAVESLGFILFR	PSMD1	26S proteasome non-ATPase regulatory subunit 1
CLDDHKYK	PSMD1	26S proteasome non-ATPase regulatory subunit 1
DKDPILRR	PSMD1	26S proteasome non-ATPase regulatory subunit 1
DNLEWLAR	PSMD1	26S proteasome non-ATPase regulatory subunit 1
DTSEDIEELVEPVAAHGPK	PSMD1	26S proteasome non-ATPase regulatory subunit 1
DTSPGSAYQEGGGLYALGLIHANHGGDIIDYLLNQLK	PSMD1	26S proteasome non-ATPase regulatory subunit 1
EAINLLEPMTNDPVNYVR	PSMD1	26S proteasome non-ATPase regulatory subunit 1
EALQLMATYLPK	PSMD1	26S proteasome non-ATPase regulatory subunit 1
FGAILAQGILDAGGHNVTISLQSR	PSMD1	26S proteasome non-ATPase regulatory subunit 1
FTATASLGVIHK	PSMD1	26S proteasome non-ATPase regulatory subunit 1
GLAVGIALVMYGR	PSMD1	26S proteasome non-ATPase regulatory subunit 1
HGGSLGLGLAAMGTAR	PSMD1	26S proteasome non-ATPase regulatory subunit 1
KKEPEPNFQLLDNPAR	PSMD1	26S proteasome non-ATPase regulatory subunit 1

LCMSLMQNK	PSMD1	26S proteasome non-ATPase regulatory subunit 1
LLHVAVSDVNDDVR	PSMD1	26S proteasome non-ATPase regulatory subunit 1
LLHVAVSDVNDDVRR	PSMD1	26S proteasome non-ATPase regulatory subunit 1
MEEADALIESLCR	PSMD1	26S proteasome non-ATPase regulatory subunit 1
MITSAAGIISLLDEDEPQLK	PSMD1	26S proteasome non-ATPase regulatory subunit 1
MITSAAGIISLLDEDEPQLKEFALHK	PSMD1	26S proteasome non-ATPase regulatory subunit 1
NAQAIEDMVGYAQETQHEK	PSMD1	26S proteasome non-ATPase regulatory subunit 1
NNNTDLMILK	PSMD1	26S proteasome non-ATPase regulatory subunit 1
NSVCHTATVIANSFMHCGTTSDQFLR	PSMD1	26S proteasome non-ATPase regulatory subunit 1
QAIGIALETR	PSMD1	26S proteasome non-ATPase regulatory subunit 1
QCVENADLPEGEKKPIDQR	PSMD1	26S proteasome non-ATPase regulatory subunit 1
QDVYDLLK	PSMD1	26S proteasome non-ATPase regulatory subunit 1
QFAALVASK	PSMD1	26S proteasome non-ATPase regulatory subunit 1
QGALIASALIMIQQTEITCPK	PSMD1	26S proteasome non-ATPase regulatory subunit 1
RLDVFEK	PSMD1	26S proteasome non-ATPase regulatory subunit 1
RLLHVAVSDVNDDVR	PSMD1	26S proteasome non-ATPase regulatory subunit 1
RSGMYTVAMAYCGSGNNK	PSMD1	26S proteasome non-ATPase regulatory subunit 1
SGMYTVAMAYCGSGNNK	PSMD1	26S proteasome non-ATPase regulatory subunit 1
SNCKPSTFAYPAPLEVPKEK	PSMD1	26S proteasome non-ATPase regulatory subunit 1
TNLYQDDAVTGEAAGLALGLVMLGSK	PSMD1	26S proteasome non-ATPase regulatory subunit 1
TPEASPEPK	PSMD1	26S proteasome non-ATPase regulatory subunit 1
TPEQCPSVVSLLSESYNPHVR	PSMD1	26S proteasome non-ATPase regulatory subunit 1
TSSAFVGK	PSMD1	26S proteasome non-ATPase regulatory subunit 1
TVGTPIASVPGSTNTGTVPGSEKDSDSMETEEK	PSMD1	26S proteasome non-ATPase regulatory subunit 1
VINDKHDDVMAK	PSMD1	26S proteasome non-ATPase regulatory subunit 1
VLTMPETCR	PSMD1	26S proteasome non-ATPase regulatory subunit 1
VMPAQLK	PSMD1	26S proteasome non-ATPase regulatory subunit 1
VSTAVLSITAK	PSMD1	26S proteasome non-ATPase regulatory subunit 1
YGAAMALGICCAGTGNK	PSMD1	26S proteasome non-ATPase regulatory

		subunit 1
YQPFKPLSIGGIIILK	PSMD1	26S proteasome non-ATPase regulatory subunit 1
DHYEATAMHR	PSMD10	26S proteasome non-ATPase regulatory subunit 10
LEELKESILADK	PSMD10	26S proteasome non-ATPase regulatory subunit 10
LLVSQGASIYIENKEEK	PSMD10	26S proteasome non-ATPase regulatory subunit 10
NRHEIAVMLLEGGANPDAK	PSMD10	26S proteasome non-ATPase regulatory subunit 10
NRHEIAVMLLEGGANPDAKDHYEATAMHR	PSMD10	26S proteasome non-ATPase regulatory subunit 10
AAAAVVEFQR	PSMD11	26S proteasome non-ATPase regulatory subunit 11
AALTSAR	PSMD11	26S proteasome non-ATPase regulatory subunit 11
AELRDDPIISTHLAK	PSMD11	26S proteasome non-ATPase regulatory subunit 11
ALLVEVQLLESK	PSMD11	26S proteasome non-ATPase regulatory subunit 11
ALTDYR	PSMD11	26S proteasome non-ATPase regulatory subunit 11
AQSLLSTDR	PSMD11	26S proteasome non-ATPase regulatory subunit 11
AQSLLSTDREASIDILHSIVK	PSMD11	26S proteasome non-ATPase regulatory subunit 11
AQSLLSTDREASIDILHSIVKR	PSMD11	26S proteasome non-ATPase regulatory subunit 11
DDPIISTHLAK	PSMD11	26S proteasome non-ATPase regulatory subunit 11
DIQENDEEAVQVK	PSMD11	26S proteasome non-ATPase regulatory subunit 11
EASIDILHSIVK	PSMD11	26S proteasome non-ATPase regulatory subunit 11
EASIDILHSIVKR	PSMD11	26S proteasome non-ATPase regulatory subunit 11
EQSILELGSLLAK	PSMD11	26S proteasome non-ATPase regulatory subunit 11
FHGILDQGEGVLIIFDEPPVDK	PSMD11	26S proteasome non-ATPase regulatory subunit 11
FHGILDQGEGVLIIFDEPPVDKTYEAALETIQNMSK	PSMD11	26S proteasome non-ATPase regulatory subunit 11
IMLNTPEDVQALVSGK	PSMD11	26S proteasome non-ATPase regulatory subunit 11
KLSQMILDK	PSMD11	26S proteasome non-ATPase regulatory subunit 11
KLSQMILDKK	PSMD11	26S proteasome non-ATPase regulatory subunit 11
LQATLDMQSGIIHAAEEKDWK	PSMD11	26S proteasome non-ATPase regulatory subunit 11
LSQMILDKK	PSMD11	26S proteasome non-ATPase regulatory subunit 11
LVSLYFDTK	PSMD11	26S proteasome non-ATPase regulatory subunit 11
LVSLYFDTKR	PSMD11	26S proteasome non-ATPase regulatory subunit 11

LYDNLLEQNLIR	PSMD11	26S proteasome non-ATPase regulatory subunit 11
RDIQENDEEAVQVK	PSMD11	26S proteasome non-ATPase regulatory subunit 11
SLADFEK	PSMD11	26S proteasome non-ATPase regulatory subunit 11
SLLDLFLDMEAATGQEVELCLECIEWAK	PSMD11	26S proteasome non-ATPase regulatory subunit 11
TGQAAELGGLLK	PSMD11	26S proteasome non-ATPase regulatory subunit 11
TTANAIYCPPK	PSMD11	26S proteasome non-ATPase regulatory subunit 11
TYHALSNLPK	PSMD11	26S proteasome non-ATPase regulatory subunit 11
VIEPFSR	PSMD11	26S proteasome non-ATPase regulatory subunit 11
VQIEHISSLIK	PSMD11	26S proteasome non-ATPase regulatory subunit 11
VVDSLYNK	PSMD11	26S proteasome non-ATPase regulatory subunit 11
YQEALHLGSQLLR	PSMD11	26S proteasome non-ATPase regulatory subunit 11
YVRPFLNSISK	PSMD11	26S proteasome non-ATPase regulatory subunit 11
AIYDTPCIQAESEK	PSMD12	26S proteasome non-ATPase regulatory subunit 12
AIYDTPCIQAESEKWQQALK	PSMD12	26S proteasome non-ATPase regulatory subunit 12
DPNNLLNDWSQK	PSMD12	26S proteasome non-ATPase regulatory subunit 12
EAASILQELQVETYGSMEKK	PSMD12	26S proteasome non-ATPase regulatory subunit 12
EEMIHNLQ	PSMD12	26S proteasome non-ATPase regulatory subunit 12
EQNGDVKEAASILQELQVETYGSMEKK	PSMD12	26S proteasome non-ATPase regulatory subunit 12
ERVEFILEQMR	PSMD12	26S proteasome non-ATPase regulatory subunit 12
FFQEENTEK	PSMD12	26S proteasome non-ATPase regulatory subunit 12
FFQEENTEKLK	PSMD12	26S proteasome non-ATPase regulatory subunit 12
GSLESPATDVFGSTEEGEKR	PSMD12	26S proteasome non-ATPase regulatory subunit 12
IYVEIER	PSMD12	26S proteasome non-ATPase regulatory subunit 12
KGSLESPATDVFGSTEEGEKR	PSMD12	26S proteasome non-ATPase regulatory subunit 12
KLEEIPK	PSMD12	26S proteasome non-ATPase regulatory subunit 12
LAGIINFQRPK	PSMD12	26S proteasome non-ATPase regulatory subunit 12
LCLAVKDYIR	PSMD12	26S proteasome non-ATPase regulatory subunit 12
LEEIPK	PSMD12	26S proteasome non-ATPase regulatory subunit 12
LFTTMELMR	PSMD12	26S proteasome non-ATPase regulatory

		subunit 12
LNSLMSLVNK	PSMD12	26S proteasome non-ATPase regulatory subunit 12
LPECAK	PSMD12	26S proteasome non-ATPase regulatory subunit 12
LQEVIETLLSLEK	PSMD12	26S proteasome non-ATPase regulatory subunit 12
MAQLLDLSVDESEAFLSNLVVNK	PSMD12	26S proteasome non-ATPase regulatory subunit 12
MEVDYSATVDQR	PSMD12	26S proteasome non-ATPase regulatory subunit 12
MEVDYSATVDQRLPECAK	PSMD12	26S proteasome non-ATPase regulatory subunit 12
MVQQCCTYVEEITDLPIK	PSMD12	26S proteasome non-ATPase regulatory subunit 12
SVVLYVILAPFDNEQSDLVHR	PSMD12	26S proteasome non-ATPase regulatory subunit 12
TASDMVSTSR	PSMD12	26S proteasome non-ATPase regulatory subunit 12
TLATIKEQNGDVK	PSMD12	26S proteasome non-ATPase regulatory subunit 12
TTHLIAKEEMIHNLQ	PSMD12	26S proteasome non-ATPase regulatory subunit 12
VDRLAGIINFQRPK	PSMD12	26S proteasome non-ATPase regulatory subunit 12
VEFILEQMR	PSMD12	26S proteasome non-ATPase regulatory subunit 12
VVEHNIR	PSMD12	26S proteasome non-ATPase regulatory subunit 12
LCLAVK	PSMD12;OR5AL1	26S proteasome non-ATPase regulatory subunit 12
AFTLGLAGLLGEGVFNFGELLMHPVLESLR	PSMD13	26S proteasome non-ATPase regulatory subunit 13
ALSVGLVK	PSMD13	26S proteasome non-ATPase regulatory subunit 13
DLPVSEQQER	PSMD13	26S proteasome non-ATPase regulatory subunit 13
DRLEFWCTDVK	PSMD13	26S proteasome non-ATPase regulatory subunit 13
FLGCVDIK	PSMD13	26S proteasome non-ATPase regulatory subunit 13
FLGCVDIKDLPVSEQQER	PSMD13	26S proteasome non-ATPase regulatory subunit 13
FYDLSSK	PSMD13	26S proteasome non-ATPase regulatory subunit 13
GSIDEVDKR	PSMD13	26S proteasome non-ATPase regulatory subunit 13
IQLLCLMEMTFTRPANHR	PSMD13	26S proteasome non-ATPase regulatory subunit 13
ITVNEVELLVMK	PSMD13	26S proteasome non-ATPase regulatory subunit 13
LEELYTK	PSMD13	26S proteasome non-ATPase regulatory subunit 13
LNIGDLQVTK	PSMD13	26S proteasome non-ATPase regulatory subunit 13
LYENFISEFEHR	PSMD13	26S proteasome non-ATPase regulatory subunit 13

MKDVPGFLQQSQNSGPGQPAVWHR	PSMD13	26S proteasome non-ATPase regulatory subunit 13
NTDRQWLIDTLYAFNSGNVER	PSMD13	26S proteasome non-ATPase regulatory subunit 13
QLTFEEIAK	PSMD13	26S proteasome non-ATPase regulatory subunit 13
QWLIDTLYAFNSGNVER	PSMD13	26S proteasome non-ATPase regulatory subunit 13
SMEMLVEHQAHDILT	PSMD13	26S proteasome non-ATPase regulatory subunit 13
SSDEAVILCK	PSMD13	26S proteasome non-ATPase regulatory subunit 13
TAWGQQPDLAANEAQLLR	PSMD13	26S proteasome non-ATPase regulatory subunit 13
TAWGQQPDLAANEAQLLRK	PSMD13	26S proteasome non-ATPase regulatory subunit 13
VHMTWVQPR	PSMD13	26S proteasome non-ATPase regulatory subunit 13
VLDLQQIK	PSMD13	26S proteasome non-ATPase regulatory subunit 13
VNPLSLVEIILHVVR	PSMD13	26S proteasome non-ATPase regulatory subunit 13
YYQTIGNHASYYK	PSMD13	26S proteasome non-ATPase regulatory subunit 13
AGVPMEVMGLMLGEFVDDYTVR	PSMD14	26S proteasome non-ATPase regulatory subunit 14
AVAVVVDPIQSVK	PSMD14	26S proteasome non-ATPase regulatory subunit 14
AVEEEDKMTPEQLAIK	PSMD14	26S proteasome non-ATPase regulatory subunit 14
EMLELAK	PSMD14	26S proteasome non-ATPase regulatory subunit 14
GKVVIDAFR	PSMD14	26S proteasome non-ATPase regulatory subunit 14
HLEEHVDVLMTSNIVQCLAAMLDTVVFK	PSMD14	26S proteasome non-ATPase regulatory subunit 14
HYYSITINYR	PSMD14	26S proteasome non-ATPase regulatory subunit 14
LGGGMPGLGQGPPTDAPAVDTAEQVYISSLALLK	PSMD14	26S proteasome non-ATPase regulatory subunit 14
LINANMMVLGHEPR	PSMD14	26S proteasome non-ATPase regulatory subunit 14
MLDMLK	PSMD14	26S proteasome non-ATPase regulatory subunit 14
MLLNLHK	PSMD14	26S proteasome non-ATPase regulatory subunit 14
MLLNLHKK	PSMD14	26S proteasome non-ATPase regulatory subunit 14
QTTSNLGHLNKPSIQALIHGLNR	PSMD14	26S proteasome non-ATPase regulatory subunit 14
SWMEGLTLQDYSEHCK	PSMD14	26S proteasome non-ATPase regulatory subunit 14
VIDVFAMPQSGTGVSVEAVDPVFQAK	PSMD14	26S proteasome non-ATPase regulatory subunit 14
VVIDAFR	PSMD14	26S proteasome non-ATPase regulatory subunit 14
AELATEEFLPVTPILEGFVILR	PSMD2	26S proteasome non-ATPase regulatory

		subunit 2
APVQPQQSPAAAPGGTDEKPSGK	PSMD2	26S proteasome non-ATPase regulatory subunit 2
AVPLALALISVSNPR	PSMD2	26S proteasome non-ATPase regulatory subunit 2
CALGVFR	PSMD2	26S proteasome non-ATPase regulatory subunit 2
CALGVFRK	PSMD2	26S proteasome non-ATPase regulatory subunit 2
DKAPVQPQQSPAAAPGGTDEKPSGK	PSMD2	26S proteasome non-ATPase regulatory subunit 2
DPNNLFMVR	PSMD2	26S proteasome non-ATPase regulatory subunit 2
DTSLYRPALEELRR	PSMD2	26S proteasome non-ATPase regulatory subunit 2
EAPADMGAHQGVAVLGIALIAMGEEIGAEMALR	PSMD2	26S proteasome non-ATPase regulatory subunit 2
ELDIMEPK	PSMD2	26S proteasome non-ATPase regulatory subunit 2
EPLLTLVK	PSMD2	26S proteasome non-ATPase regulatory subunit 2
EWQELDDAEK	PSMD2	26S proteasome non-ATPase regulatory subunit 2
EWQELDDAEKVQR	PSMD2	26S proteasome non-ATPase regulatory subunit 2
EWQELDDAEKVQREPLLTLVK	PSMD2	26S proteasome non-ATPase regulatory subunit 2
FAADIISVLAMTMSGER	PSMD2	26S proteasome non-ATPase regulatory subunit 2
FAADIISVLAMTMSGERECLK	PSMD2	26S proteasome non-ATPase regulatory subunit 2
FGGSGSQVDSAR	PSMD2	26S proteasome non-ATPase regulatory subunit 2
FLRPHYGK	PSMD2	26S proteasome non-ATPase regulatory subunit 2
FSRFPEALR	PSMD2	26S proteasome non-ATPase regulatory subunit 2
GEAIEAILAALEVVSEPFR	PSMD2	26S proteasome non-ATPase regulatory subunit 2
GTLTLCPYHSDR	PSMD2	26S proteasome non-ATPase regulatory subunit 2
LAAMLR	PSMD2	26S proteasome non-ATPase regulatory subunit 2
LALMLNDMELVEDIFTSCK	PSMD2	26S proteasome non-ATPase regulatory subunit 2
LAQGLTHLGK	PSMD2	26S proteasome non-ATPase regulatory subunit 2
LGSIFGLGLAYAGSNR	PSMD2	26S proteasome non-ATPase regulatory subunit 2
LGSIFGLGLAYAGSNREDVLTLLLPVMGDSK	PSMD2	26S proteasome non-ATPase regulatory subunit 2
LKEIYENMAPGENKR	PSMD2	26S proteasome non-ATPase regulatory subunit 2
LLTDDGNK	PSMD2	26S proteasome non-ATPase regulatory subunit 2
LLTDDGNKWLYK	PSMD2	26S proteasome non-ATPase regulatory subunit 2

LNILDTLSK	PSMD2	26S proteasome non-ATPase regulatory subunit 2
LVGSQEELASWGHEYVR	PSMD2	26S proteasome non-ATPase regulatory subunit 2
MLVTFDEELRPLPVSVR	PSMD2	26S proteasome non-ATPase regulatory subunit 2
MNLASSFVNGFVNAAFGQDK	PSMD2	26S proteasome non-ATPase regulatory subunit 2
NECDPALALLSDYVLHNSNTMR	PSMD2	26S proteasome non-ATPase regulatory subunit 2
QMAFMLGR	PSMD2	26S proteasome non-ATPase regulatory subunit 2
SETELKDTYAR	PSMD2	26S proteasome non-ATPase regulatory subunit 2
SFANTLVDVCAYAGSGNVLK	PSMD2	26S proteasome non-ATPase regulatory subunit 2
SGALLACGIVNSGVR	PSMD2	26S proteasome non-ATPase regulatory subunit 2
SHYVLYGLVAAMQPR	PSMD2	26S proteasome non-ATPase regulatory subunit 2
SSMEVAGVTALACGMIAVGSCNGDVTSTILQTIMEK	PSMD2	26S proteasome non-ATPase regulatory subunit 2
SSTTSMTSVPKPLK	PSMD2	26S proteasome non-ATPase regulatory subunit 2
TFGHLLR	PSMD2	26S proteasome non-ATPase regulatory subunit 2
TITGFQTHTTPVLLAHGER	PSMD2	26S proteasome non-ATPase regulatory subunit 2
VCLYLTSCVNYVPEPENSALLR	PSMD2	26S proteasome non-ATPase regulatory subunit 2
VGQAVDVVGQAGKPK	PSMD2	26S proteasome non-ATPase regulatory subunit 2
VPDDIYKTHLENNR	PSMD2	26S proteasome non-ATPase regulatory subunit 2
VQQLLHICSEHFDSK	PSMD2	26S proteasome non-ATPase regulatory subunit 2
VQREPLLTLVK	PSMD2	26S proteasome non-ATPase regulatory subunit 2
YGEPTLR	PSMD2	26S proteasome non-ATPase regulatory subunit 2
YGEPTLRR	PSMD2	26S proteasome non-ATPase regulatory subunit 2
YLYSSEDYIK	PSMD2	26S proteasome non-ATPase regulatory subunit 2
FPEALR	PSMD2;ITGAM	26S proteasome non-ATPase regulatory subunit 2
AASTPLLPEVEAYLQLLVVIFMMNSK	PSMD3	26S proteasome non-ATPase regulatory subunit 3
AASTPLLPEVEAYLQLLVVIFMMNSKR	PSMD3	26S proteasome non-ATPase regulatory subunit 3
AIQLEYSEAR	PSMD3	26S proteasome non-ATPase regulatory subunit 3
AIQLEYSEARR	PSMD3	26S proteasome non-ATPase regulatory subunit 3
AIRDGVIEASINHEK	PSMD3	26S proteasome non-ATPase regulatory subunit 3
AKPPPGGGEQEPPPPPAPQDVEMK	PSMD3	26S proteasome non-ATPase regulatory

		subunit 3
AKPPPGGGEQEPPPPPAPQDVEMKEEAATGGGSTGE ADGK	PSMD3	26S proteasome non-ATPase regulatory subunit 3
ALDLVAAK	PSMD3	26S proteasome non-ATPase regulatory subunit 3
APQHTAVGFK	PSMD3	26S proteasome non-ATPase regulatory subunit 3
AVQGFFTSNNATR	PSMD3	26S proteasome non-ATPase regulatory subunit 3
CYYYHAR	PSMD3	26S proteasome non-ATPase regulatory subunit 3
DFLLPFLEEPMDTEADLQFRPR	PSMD3	26S proteasome non-ATPase regulatory subunit 3
DGVIEASINHEK	PSMD3	26S proteasome non-ATPase regulatory subunit 3
DLESAEER	PSMD3	26S proteasome non-ATPase regulatory subunit 3
DLESAEERR	PSMD3	26S proteasome non-ATPase regulatory subunit 3
ELDTVTLEDIKEHVK	PSMD3	26S proteasome non-ATPase regulatory subunit 3
EMIDIYSTR	PSMD3	26S proteasome non-ATPase regulatory subunit 3
EMIDIYSTREPQLAFHQR	PSMD3	26S proteasome non-ATPase regulatory subunit 3
EPQLAFHQR	PSMD3	26S proteasome non-ATPase regulatory subunit 3
EQQDLEFAK	PSMD3	26S proteasome non-ATPase regulatory subunit 3
FNQVLDQFGEK	PSMD3	26S proteasome non-ATPase regulatory subunit 3
FNQVLDQFGEKFQADGTYTLIIR	PSMD3	26S proteasome non-ATPase regulatory subunit 3
FQADGTYTLIIR	PSMD3	26S proteasome non-ATPase regulatory subunit 3
HDADGQATLLNLLLR	PSMD3	26S proteasome non-ATPase regulatory subunit 3
ISDDLMQK	PSMD3	26S proteasome non-ATPase regulatory subunit 3
ISFCLDIHNMSVK	PSMD3	26S proteasome non-ATPase regulatory subunit 3
ISLADIAQK	PSMD3	26S proteasome non-ATPase regulatory subunit 3
KAPQHTAVGFK	PSMD3	26S proteasome non-ATPase regulatory subunit 3
LLIVVELLLGEIPDR	PSMD3	26S proteasome non-ATPase regulatory subunit 3
LLIVVELLLGEIPDRLQFR	PSMD3	26S proteasome non-ATPase regulatory subunit 3
LNHYVLYK	PSMD3	26S proteasome non-ATPase regulatory subunit 3
LQLDSPEDAEFIVAK	PSMD3	26S proteasome non-ATPase regulatory subunit 3
MISLSYSR	PSMD3	26S proteasome non-ATPase regulatory subunit 3
NYLHYSLYDQAEK	PSMD3	26S proteasome non-ATPase regulatory subunit 3

RALDLVAAK	PSMD3	26S proteasome non-ATPase regulatory subunit 3
RLNHYVLYK	PSMD3	26S proteasome non-ATPase regulatory subunit 3
RTMTNALR	PSMD3	26S proteasome non-ATPase regulatory subunit 3
SFLHAR	PSMD3	26S proteasome non-ATPase regulatory subunit 3
SLMPYFLLTQAVR	PSMD3	26S proteasome non-ATPase regulatory subunit 3
SVFPEQANNNEWAR	PSMD3	26S proteasome non-ATPase regulatory subunit 3
SYNKDLESAEER	PSMD3	26S proteasome non-ATPase regulatory subunit 3
SYNKDLESAEERR	PSMD3	26S proteasome non-ATPase regulatory subunit 3
TAAAAAEHSQRELDTVTLEDIKEHVK	PSMD3	26S proteasome non-ATPase regulatory subunit 3
TMTNALR	PSMD3	26S proteasome non-ATPase regulatory subunit 3
VYEFLDKLDVVR	PSMD3	26S proteasome non-ATPase regulatory subunit 3
YLYYTGR	PSMD3	26S proteasome non-ATPase regulatory subunit 3
AAAASAAEAGIATTGTEDSDDALLK	PSMD4	26S proteasome non-ATPase regulatory subunit 4
ITFCTGIR	PSMD4	26S proteasome non-ATPase regulatory subunit 4
LQAQQDAVNIVCHSK	PSMD4	26S proteasome non-ATPase regulatory subunit 4
MTISQQEFGR	PSMD4	26S proteasome non-ATPase regulatory subunit 4
NAMGSLASQATK	PSMD4	26S proteasome non-ATPase regulatory subunit 4
RAAAASAAEAGIATTGTEDSDDALLK	PSMD4	26S proteasome non-ATPase regulatory subunit 4
AEYLCR	PSMD6	26S proteasome non-ATPase regulatory subunit 6
ANEDELK	PSMD6	26S proteasome non-ATPase regulatory subunit 6
DELMAAVR	PSMD6	26S proteasome non-ATPase regulatory subunit 6
DNNMAPYYEALCK	PSMD6	26S proteasome non-ATPase regulatory subunit 6
DWLFAPHYR	PSMD6	26S proteasome non-ATPase regulatory subunit 6
EGALTAFR	PSMD6	26S proteasome non-ATPase regulatory subunit 6
EGALTAFRK	PSMD6	26S proteasome non-ATPase regulatory subunit 6
FIAAGR	PSMD6	26S proteasome non-ATPase regulatory subunit 6
FLLSLPEHR	PSMD6	26S proteasome non-ATPase regulatory subunit 6
GAEILEVLHSLPAVR	PSMD6	26S proteasome non-ATPase regulatory subunit 6
GDAAVRDELMAAVR	PSMD6	26S proteasome non-ATPase regulatory

		subunit 6
GDLLLNR	PSMD6	26S proteasome non-ATPase regulatory subunit 6
IDKVNEIVETNRPDSK	PSMD6	26S proteasome non-ATPase regulatory subunit 6
IGDKEGALTAFR	PSMD6	26S proteasome non-ATPase regulatory subunit 6
IGDKEGALTAFRK	PSMD6	26S proteasome non-ATPase regulatory subunit 6
IGLFYMDNDLITR	PSMD6	26S proteasome non-ATPase regulatory subunit 6
IHAYSQLLESYR	PSMD6	26S proteasome non-ATPase regulatory subunit 6
KDWLFAPHYR	PSMD6	26S proteasome non-ATPase regulatory subunit 6
KGDLLLNR	PSMD6	26S proteasome non-ATPase regulatory subunit 6
LDEELEDAEKNLGESEIR	PSMD6	26S proteasome non-ATPase regulatory subunit 6
LDIVFYLLR	PSMD6	26S proteasome non-ATPase regulatory subunit 6
LKVYQGLYCVAIR	PSMD6	26S proteasome non-ATPase regulatory subunit 6
NLGESEIR	PSMD6	26S proteasome non-ATPase regulatory subunit 6
NWQYQETIK	PSMD6	26S proteasome non-ATPase regulatory subunit 6
NWQYQETIKK	PSMD6	26S proteasome non-ATPase regulatory subunit 6
PLENLEEEGLPK	PSMD6	26S proteasome non-ATPase regulatory subunit 6
PLENLEEEGLPKNPDLR	PSMD6	26S proteasome non-ATPase regulatory subunit 6
QAAELFLDTVSTFTSYELMDYK	PSMD6	26S proteasome non-ATPase regulatory subunit 6
QYLFSLYECR	PSMD6	26S proteasome non-ATPase regulatory subunit 6
RLDEELEDAEKNLGESEIR	PSMD6	26S proteasome non-ATPase regulatory subunit 6
SLDWQIDVDLLNK	PSMD6	26S proteasome non-ATPase regulatory subunit 6
SLIEEGGDWDR	PSMD6	26S proteasome non-ATPase regulatory subunit 6
SLIEEGGDWDRR	PSMD6	26S proteasome non-ATPase regulatory subunit 6
SLTLGYMAEAFGVGVEFIDQELSR	PSMD6	26S proteasome non-ATPase regulatory subunit 6
TFVTYTVYVSMIALERPDLR	PSMD6	26S proteasome non-ATPase regulatory subunit 6
VNEIVETNRPDSK	PSMD6	26S proteasome non-ATPase regulatory subunit 6
VYQGLYCVAIR	PSMD6	26S proteasome non-ATPase regulatory subunit 6
YSVFFQSLAVVEQEMK	PSMD6	26S proteasome non-ATPase regulatory subunit 6
YSVFFQSLAVVEQEMKK	PSMD6	26S proteasome non-ATPase regulatory subunit 6

DIKDTTVGTLSQR	PSMD7	26S proteasome non-ATPase regulatory subunit 7
DLGLPTEAYISVEEVHDDGTPTSK	PSMD7	26S proteasome non-ATPase regulatory subunit 7
DTTVGTLSQR	PSMD7	26S proteasome non-ATPase regulatory subunit 7
ITNQVHGLK	PSMD7	26S proteasome non-ATPase regulatory subunit 7
IVGWYHTGPK	PSMD7	26S proteasome non-ATPase regulatory subunit 7
LHKNDIAINELMK	PSMD7	26S proteasome non-ATPase regulatory subunit 7
LHKNDIAINELMKR	PSMD7	26S proteasome non-ATPase regulatory subunit 7
LPINHQIIYQLQDVFNLLPDVSLQEFVK	PSMD7	26S proteasome non-ATPase regulatory subunit 7
NDIAINELMK	PSMD7	26S proteasome non-ATPase regulatory subunit 7
PELAVQK	PSMD7	26S proteasome non-ATPase regulatory subunit 7
RVVGVLLGSWQK	PSMD7	26S proteasome non-ATPase regulatory subunit 7
SVVALHNLINNK	PSMD7	26S proteasome non-ATPase regulatory subunit 7
TFEHVTSEIGAEEAEEVGVEHLLR	PSMD7	26S proteasome non-ATPase regulatory subunit 7
TNDQMVVVYLASLIR	PSMD7	26S proteasome non-ATPase regulatory subunit 7
VVGVLLGSWQK	PSMD7	26S proteasome non-ATPase regulatory subunit 7
VVGVLLGSWQKK	PSMD7	26S proteasome non-ATPase regulatory subunit 7
VVVHPLVLLSVVDHFNR	PSMD7	26S proteasome non-ATPase regulatory subunit 7
YCPNSVLVIIDVKPK	PSMD7	26S proteasome non-ATPase regulatory subunit 7
DIQTNVYIK	PSMD8	26S proteasome non-ATPase regulatory subunit 8
ILFFNTPK	PSMD8	26S proteasome non-ATPase regulatory subunit 8
ILFTEATR	PSMD8	26S proteasome non-ATPase regulatory subunit 8
VAEFHTELER	PSMD8	26S proteasome non-ATPase regulatory subunit 8
AMLRVQPEAQAK	PSME1	Proteasome activator complex subunit 1
DKKDEEEDMSLD	ADRM1	Proteasomal ubiquitin receptor ADRM1
GLVYIQQTDDSLIHFCWK	ADRM1	Proteasomal ubiquitin receptor ADRM1
LFFWMQEPK	ADRM1	Proteasomal ubiquitin receptor ADRM1
RLFFWMQEPK	ADRM1	Proteasomal ubiquitin receptor ADRM1
SQSAAVTPSSTTSSTR	ADRM1	Proteasomal ubiquitin receptor ADRM1
YLVEFR	ADRM1	Proteasomal ubiquitin receptor ADRM1
