# Supplementary figures and images for: Golgi organization is regulated by proteasomal degradation
Source: Nat Commun. 2020 Jan 21;11:409. doi: 10.1038/s41467-019-14038-9 (PMC6972958; doi:10.1038/s41467-019-14038-9)

Figure 2G

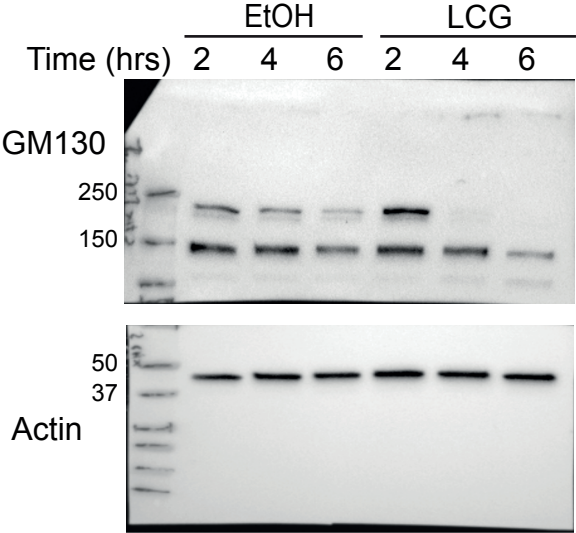

Figure 4B

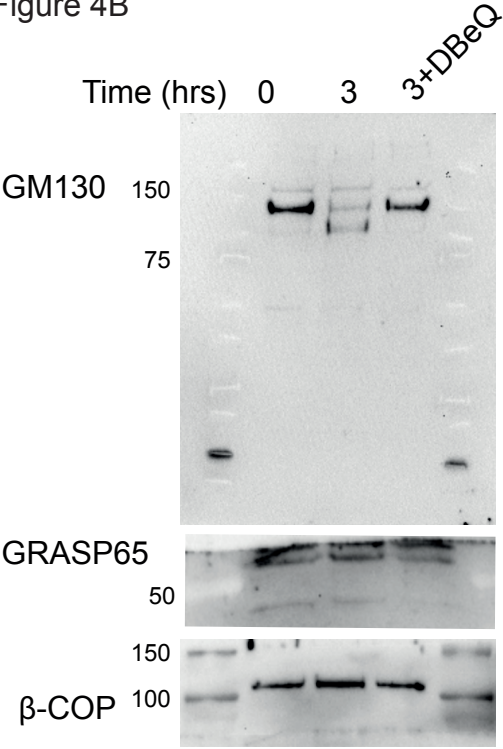

Figure 1H

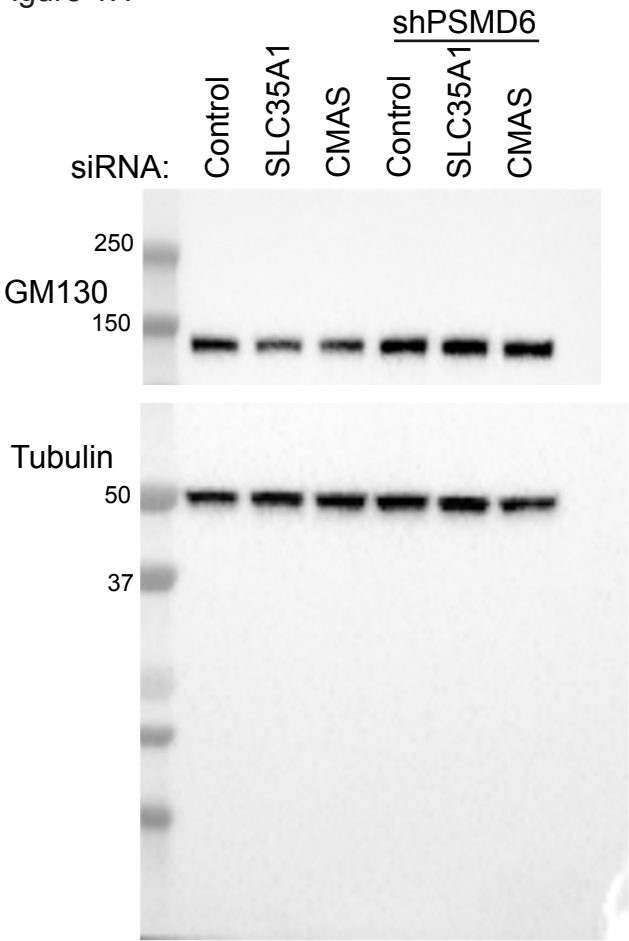

Figure 3F

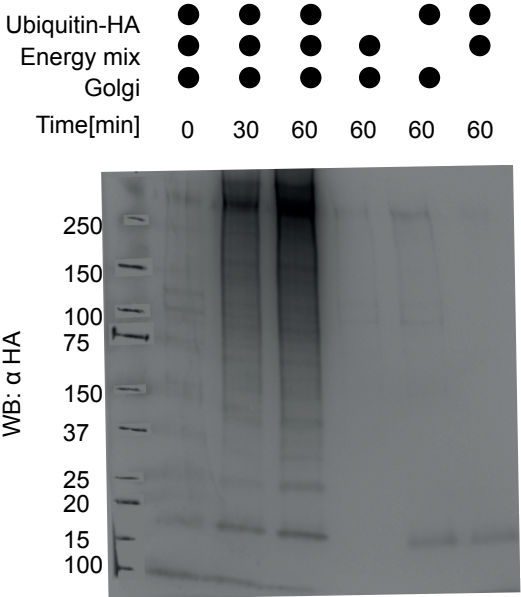

Supplement: Supplementary file 4 — Source Data [file 41467_2019_14038_MOESM4_ESM.pdf]
